# Transcriptomic and metabolic regulatory network characterization of drought responses in tobacco

**DOI:** 10.3389/fpls.2022.1067076

**Published:** 2023-01-18

**Authors:** Zhengrong Hu, Zexue He, Yangyang Li, Qing Wang, Pengfei Yi, Jiashuo Yang, Chenkai Yang, Gennadii Borovskii, Xuejiao Cheng, Risheng Hu, Wenli Zhang

**Affiliations:** ^1^ Hunan Tobacco Research Institute, Changsha, Hunan, China; ^2^ State Key Laboratory for Crop Genetics and Germplasm Enhancement, Jiangsu Collaborative Innovation Center for Modern Crop Production (JCIC-MCP), Collaborative Innovation Center for Modern Crop Production Co-Sponsored by Province and Ministry (CIC-MCP), Nanjing Agricultural University, Nanjing, Jiangsu, China; ^3^ Hu'nan Tobacco Company Changde Company, Changde, Hunan, China; ^4^ College of Agronomy, Hunan Agricultural University, Changsha, Hunan, China; ^5^ Siberian Institute of Plant Physiology and Biochemistry Siberian Branch of Russian Academy of Sciences (SB RAS) Irkutsk, Lermontova, Russia

**Keywords:** drought stress, transcriptome, metabolome, regulatory network, *Nicotiana tabacum* L.

## Abstract

Drought stress usually causes huge economic losses for tobacco industries. Drought stress exhibits multifaceted impacts on tobacco systems through inducing changes at different levels, such as physiological and chemical changes, changes of gene transcription and metabolic changes. Understanding how plants respond and adapt to drought stress helps generate engineered plants with enhanced drought resistance. In this study, we conducted multiple time point-related physiological, biochemical,transcriptomic and metabolic assays using K326 and its derived mutant 28 (M28) with contrasting drought tolerance. Through integrative analyses of transcriptome and metabolome,we observed dramatic changes of gene expression and metabolic profiles between M28 and K326 before and after drought treatment. we found that some of DEGs function as key enzymes responsible for ABA biosynthesis and metabolic pathway, thereby mitigating impairment of drought stress through ABA signaling dependent pathways. Four DEGs were involved in nitrogen metabolism, leading to synthesis of glutamate (Glu) starting from NO−3 /NO−2 that serves as an indicator for stress responses. Importantly, through regulatory network analyses, we detected several drought induced TFs that regulate expression of genes responsible for ABA biosynthesis through network, indicating direct and indirect involvement of TFs in drought responses in tobacco. Thus, our study sheds some mechanistic insights into how plant responding to drought stress through transcriptomic and metabolic changes in tobacco. It also provides some key TF or non-TF gene candidates for engineering manipulation for breeding new tobacco varieties with enhanced drought tolerance.

## Introduction

Drought stress is one of key restricting environmental factors that adversely affect plant growth and development. In particular, it dramatically reduces global crop productivity in arid and semi-arid regions, depending on its severity and duration (Kumar et al., 2018). It attracts more attention worldwide with the increasing severity of global warming. Drought stress exhibits multifaceted impacts on plant systems through inducing changes at different levels, such as physiological and chemical changes, changes of gene transcription and epigenetic changes (Chang et al., 2020; Li et al., 2021; Hura et al., 2022). For instance, it causes small plant architectures or plant death (Liu et al., 2017; Romdhane et al., 2020) and reactive oxygen species (ROS) related cell or membrane damage (Cruz de Carvalho, 2008; Abid et al., 2018; Couchoud et al., 2019); decreases transpiration through reducing stomatal conductance or stomatal closure (Li et al., 2017), and photosynthetic performance (Cornic, 2000); alters water use efficiency (WUE) (Karaba et al., 2007; Liu et al., 2017), phytohormone levels and distributions (Gupta et al., 2017; Tiwari et al., 2017; Pavlovic et al., 2018), changes of antioxidant or other enzyme activities (Abid et al., 2018; Laxa et al., 2019); alters transcription of transcription factors (TFs), non-TF genes, or non-coding RNAs (ncRNAs) (Sakuma et al., 2006; Harb et al., 2010; Guo et al., 2013; Huang et al., 2018; Yuan et al., 2018; Li et al., 2019b; Roca Paixao et al., 2019; Tang et al., 2019; Wang et al., 2020; Weidong et al., 2020). Moreover, it has been documented that drought stress can affect accumulation of different osmolytes or metabolites such as glycinebetaine, organic acids, polyols, polyamines, proline (Capell et al., 2004; Quan et al., 2004; Zhang et al., 2017; Abid et al., 2018; Michaletti et al., 2018; Pal et al., 2018). Thus, plants usually experience multiple changes on exposure to drought stress, including morphological, cellular, physiological, biochemical, transcriptional, epigenetic and metabolic alterations (Farooq et al., 2009; Xu et al., 2010; Fathi and Barari, 2016; Joshi et al., 2016; Hussain et al., 2018; Kapoor et al., 2020).

Understanding how plants respond and adapt to drought stress helps to generate bioengineered plants with enhanced drought resistance, which is an urgent need for crops, as the global warming has become the subject of debate worldwide. Transcriptional changes are one of the underlying molecular mechanisms responsible for the subsequent physiological, biochemical and metabolic alterations under the drought conditions. Therefore, transcriptional analyses of key TFs and non-TF genes involved in drought responses have been extensively explored in multiple plant species, including rice (Degenkolbe et al., 2009; Moumeni et al., 2015; Chung et al., 2016), maize (Cao et al., 2018; Zenda et al., 2019), wheat (Aprile et al., 2009; Liu et al., 2018), peal millet (Shivhare et al., 2020), sorghum (Varoquaux et al., 2019), *Brassica rapa* (Greenham et al., 2017), tomato (Gong et al., 2010), tea (Parmar et al., 2019), and tobacco (Rabara et al., 2015; Yin et al., 2015; Yang et al., 2017). In general, global transcriptional landscapes across various species provide an overview regarding how drought-inducible genes are involved in drought responses or adaptation in plants, thereby facilitating identification of key genes for further validation or applications.

In addition to be as an economic commercial non-food crop worldwide (Hu et al., 2006), tobacco (*Nicotiana tabacum* L.) is also a popular model plant for functional genomic studies (Elleuch et al., 2001; Rushton et al., 2008). Unfortunately, tobacco production in arid and semi-arid regions worldwide occasionally suffers from drought stress (Yang et al., 2017), inevitably leading to huge economic losses for tobacco growers and industries. Breeding and cultivation of bioengineered tobacco varieties with enhanced drought resistance will be a feasible way to avoid economic loss for tobacco production. Currently, mechanisms related to drought resistance in tobacco have been investigated at transcriptional, biochemical and metabolic levels (Rizhsky et al., 2002; Rabara et al., 2015; Rabara et al., 2017; Su et al., 2017; Yang et al., 2017; Chen et al., 2019), providing some valuable information to guide tobacco breeding. However, comprehensive transcriptional and metabolic network analyses of wild type tobacco and its derived mutant with contrasting drought responses are still not well studied.

In this study, we conducted transcriptional and metabolic analyses using K326 and its derived mutant 28 (M28) with contrasting drought tolerance. Through integration of RNA-seq with metabolic data, and construction of gene regulatory networks, we revealed that some key genes directly or indirectly regulate differential gene expression or accumulation of metabolites potentially involved in drought responses.

## Materials and methods

### Plant growth and drought treatment

Seeds of K326 and its derived EMS mutant, M28 with enhanced drought resistance, were sterilized and pretreated at 4°C for 2 days. The pretreated seeds were put into pots with nutrient soil for germination. The germinated seedlings with fully expanded cotyledons were transferred into small pots. Each pot contained the exactly same amount (120g) of nutrient soil, 10 seedlings per pot for each replicate, total three replicates for K326 or M28. All pots were randomly put into the incubator to grow under 22 ± 1°C,16h/8h light-dark cycle and 70% humidity. At the fully expanded five-leaf stage, each pot with 3 similar-sized seedlings was fully watered, and then all pots with K326 or M28 were randomly divided into 3 parts for drought treatment, natural drought (ND) treatment and 200 mM mannitol for simulated drought (SD) treatment. According to visible phenotypic changes (4h/24h for SD and 7D/14D for ND), we decided to collect the third leaf of each plant from the top to the bottom at 7 and 14 days after ND, or at 1h and 4h post SD for the downstream physiological and biochemical assays. Considering that drought induced transcriptional changes occur prior to occurrence of phenotypic changes, we decided to collect leaves with ND and SD and the corresponding CK at 0, 1, 2, 4 and 8h, respectively, for the downstream RNA-seq and metabolome analyses.

### Physiological and biochemical assays

**Leaf water content assay**: The collected leaves from drought treatment and CK were measured with a balance for fresh weight (FW) followed by merging the leaves into ddH2O for 6-8h for maximizing turgidity. Completely turgid leaves were measured with a balance for saturated fresh weight (SFW) followed by oven-dried at 80 °C for 24 h for measuring dry weight (DW). Relative leaf water content (RWC) and water saturation deficit (WSD) were calculated according to the formulas:


RWC=(FW−DW)/(SFW−DW); WSD =(SFW − FW)/(SFW−DW)

**Relative conductivity:** The leaves were submerged into 10 ml ddH_2_O in the centrifuge tube, and constantly shaking at RT for 24 h. The first conductivity (A) was measured followed by heating to 95 °C for 15 min, after cooling down to RT, the second conductivity (B) was measured. The relative conductivity was calculated as A/B*100%.

**Net photosynthetic rate, Stomatal conductance and Transpiration rate:** Those three parameters were measured and recorded by using portable photosynthesis system (LI-6400XT, LI-COR, Inc., Lincoln, NE, USA), which was conducted from 9:30 AM to 11:00 AM at 26°C and photosynthetic photon flux as 600 μmol·m^-2^·s^-1^.

**Enzyme activity assays:** Firstly, 0.2 g of fully expanded functional leaves were weighed and ground into powder using liquid nitrogen. 4 mL of pre-cooled 150 mmol/L phosphate buffer solution (pH 7.0) was added to mix well, and the homogenate was transferred into a 15 mL centrifuge tube. After centrifugation at 12,000 rpm at 4 °C for 20 min, the supernatant was collected and used as the crude enzyme solution. MDA content and POD activity were determined according to the method described in Hu (Hu et al., 2016). SOD activity and H_2_O_2_ content were measured using SOD assay kit (A001-3-2, Nanjing Jiancheng Bioengineering Institute, China) and Hydrogen Peroxide assay kit (A064-1-1, Nanjing Jiancheng Bioengineering Institute, China) respectively, according to the manufacturer’s instructions.

### Virus-induced Gene silencing of the target genes

For construction of the recombinant of pTRV2: NCED1, a fragment of about 300 bp of NCED1 was cloned and inserted into the restriction site of rDNA11 of pTRV2. pTRV2: PP2C-37L and pTRV2: P450 84A1L were constructed by using the same method. pTRV1 and the three vectors containing the target gene sequences were then transformed into a bacteria A. tumefaciens strain GV3101, respectively.

Tumefaciens positive clones were selected for culture in 500 μL LB liquid medium (containing 50 μg/ml kana and 25 μg/ml rifampicin) with constant shaking at 200 rpm, at 28°C overnight. They were further cultured with 50 mL the same LB medium with constant shaking at 28°C for 16-24 h with OD600 reaching about 0.6-0.8. The thalli were collected by centrifugation (4,000 rpm for 20 min) and treated with infection buffer (10 mM MES; 10 mm MgCl2; 200 μm AS), adjusted to OD600 between 1.0-1.5. The Agrobacterium solution with equal concentration of pTRV1 and pTRV2 (pTRV2: NCED1, pTRV2: PP2C-37L, pTRV2: P450 84A1L) were mixed and left at room temperature for 3 h. A syringe without a needle was used to inject 400-600 μL of the prepared infection solution into the back of the leaf from 25-day-old tobacco (*Nicotiana benthamiana*) for infection. After 24-hour dark acclimation, inoculated tobacco plants were transferred to normal culture conditions (25°C, 240 μmol m-2s-1wih 16 h/8 h light/dark cycle) for 14 days. They were then subjected to drought stress treatment for 14 days, after which it was rehydrated. The leaf samples were collected at 7 days of drought stress for RNA extraction.

RNA extraction and reverse transcription were performed using the kits (Norvezan RC401, Norvezan R312-01) according to the instructions. qRT-PCR was conducted by using fluorescent intercalating dye PowerUp™ SYBR™ Green Master Mix (Thermo Fisher Scientific, 100029284) with the QuantStudio™ 3 Real-Time PCR Instrument (Applied Biosystems, USA). The gene-specific primers are listed in **Supplementary Table S10**. The 18S rDNA was used as the internal standard gene. Each sample was biologically triplicated.

### RNA-seq and data analyses

Total RNA was extracted using collected leaves with ND and SD at 0, 1, 2, 4 and 8h. After completely removal of genomic DNA contamination, mRNA was enriched for RNA-seq library preparation following the kit instruction.

For RNA-seq analyses, the quality of raw reads was processed using FastQC (Kroll et al., 2014). Adapter contamination and poor-quality bases were removed using Trimmomatic (version 0.39) (Bolger et al., 2014). Hisat2 (version 2.1.0) (Kim et al., 2015) was used to align clean reads to the tobacco reference genome. FeatureCounts (version 1.6.4) (Yan et al., 2014) was used for counting number of reads and FPKM (Fragments Per Kilobase of exon model per Million mapped fragments) values, representing gene expression levels. DESeq2 R package (version 1.30.1) (Varet et al., 2016) was used for identification of differentially expressed genes (DEGs) between M28 and K326. DGEs were determined with a cutoff as padj<0.01 and $\left|{\text{log}}_{2}^{\text{FoldChange}}\right|$ >1.

### Gene Ontology and KEGG analyses

Gene Ontology (GO) and KEGG analyses of DEGs were analyzed using AgriGO (Tian et al., 2017) and KOBAS (version3.0) (Bu et al., 2021), respectively.

### UPLC-MS/MS metabolite measurements and data analyses

The freeze-dried samples were crushed using a mixer mill (MM 400, Retsch) with a zirconia bead for 1.5 min at 30 Hz. 100 mg of lyophilized powder per sample was dissolved using 1.2 ml of 70% methanol solution, vortexed 30 seconds every 30 minutes for 6 times in total. The homogenized samples were placed in a refrigerator at 4°C overnight. After centrifugation at 12,000rpm for 10 min, the extracts were filtrated using millipore filter (0.22 μm pore size). The filtrates were analyzed using an UPLC-ESI-MS/MS system. The analytical conditions were conducted as follows: UPLC: column, Agilent SB-C18 (1.8 µm, 2.1 mm*100 mm), the mobile phase consisting of solvent A (pure water with 0.1% formic acid) and solvent B (acetonitrile with 0.1% formic acid). Sample measurements were performed with a gradient program as below: the starting conditions as 95% A, 5% B, then a programmed linear gradient as 5% A, 95% B within 9 min, and a composition of 5% A, 95% B kept for 1 min; a composition of 95% A, 5.0% B subsequently adjusted within 1.10 min and kept for 2.9 min; the flow velocity set as 0.35ml per minute; the column oven set to 40°C. The injection volume was 4 μl. The effluent was alternatively connected to an ESI-triple quadrupole-linear ion trap (QTRAP)-MS. LIT and triple quadrupole (QQQ) scans were acquired on a triple quadrupole-linear ion trap mass spectrometer (Q TRAP), AB4500 Q TRAP UPLC/MS/MS System, equipped with an ESI Turbo Ion-Spray interface, which was operated in positive and negative ion mode and controlled by Analyst 1.6.3 software (AB Sciex). The ESI source operation parameters were as follows: ion source, turbo spray; source temperature 550°C; ion spray voltage (IS) 5500 V (positive ion mode)/-4500 V (negative ion mode); ion source gas I (GSI), gas II (GSII) and curtain gas (CUR) were set at 50, 60, and 25.0 psi, respectively; the collision-activated dissociation (CAD) was high. Instrument tuning and mass calibration were performed with 10 and 100 μmol/L polypropylene glycol solutions in QQQ and LIT modes, respectively. QQQ scans were acquired as MRM experiments with collision nitrogen gas set to medium. DP and CE for individual MRM transitions were done with further DP and CE optimization. A specific set of MRM transitions were monitored for each period according to the metabolites eluted within this period

Drought induced differential metabolic changes in M28 or K326 or between M28 and K326 were analyzed using MetaboAnalyst5.0 (Pang et al., 2021). PCA and orthogonal partial least squares discrimination analysis (OPLS-DA) were performed for unsupervised multivariate statistical analyses and supervised analyses, respectively, and to identify important variables with discriminative power. VIP is the weighted sum of the squares of the OPLS-DA analyses, indicating the importance of a variable to the entire model. The significantly differential metabolites were determined based on the combinations of a statistically significant threshold of a VIP > 1.0, a Student’s t-test p < 0.05 and $\left|{\text{log}}_{2}^{\text{FoldChange}}\right|$ > 1.

### Co-expression network analyses

The FPKM matrix of gene expression was used as input data for filtering the genes with low variations of expression levels among 30 samples according to the median absolute deviation (MAD) value > 0.5 for each gene. The remaining 14,445 genes were used to construct the weighted gene co-expression network using the R package WGCNA (v1.69) (Langfelder and Horvath, 2008). The best-weighted coefficient (*β* = 14) was determined by using the function pickSoftThreshold in the R package. The default parameters were used to construct the scale-free network. The module Hub genes were identified using the threshold as |kME| > 0.8.

### Construction of predicted TF regulatory networks

TF centered regulatory networks were predicted using all sample gene expression matrix exprMatr as input argument, which was conducted using the R package GENIE3 (v1.12.0) (Huynh-Thu and Geurts, 2018). The predicted TF regulatory networks were constructed using the three TFs in the blue co-expressed module. The DGEs within the predicted TF regulatory networks were used as potential regulatory genes, which were extracted according to the calculated “weight” values (≥ 0, a measure of the regulatory confidence level as defined by GENIE3). All TF-centered regulatory networks are visualized using Cytoscape (v3.7.2) software.

## Results

### Differential morphological, physiological and biochemical changes between K326 and M28 during drought stress

Through EMS (Ethylmethanesulfonate) treatment following by subsequent screening, we obtained one mutant, named as M28, with increased tolerance to drought stress as compared to the wild type K326. To assess drought responses between K326 and M28, we grew them in the growth chamber, three plants per pot, for two weeks followed by progressive natural drought (ND) stress without watering. As shown in **Supplementary Figure S1**, leaves of both plants became markedly wilt started at 14D post ND. For instance, M28 had more green leaves than K326 at 31 D post ND. At 3D post re-watering, three M28 and one K326 plants were recovered, respectively, indicating that M28 is more drought resistance than K326. We also applied 200 mM mannitol to simulate the drought conditions (simulated drought stress, SD). As illustrated in **Supplementary Figure S2**, K326 had visible droop leaves starting at 4h post SD, and the number of droop leaves increased with a longer duration, like from 8h to 48h post SD, by contrast, there were almost no visible morphological changes in leaves of M28 with SD at all time points.

**Figure 1:**
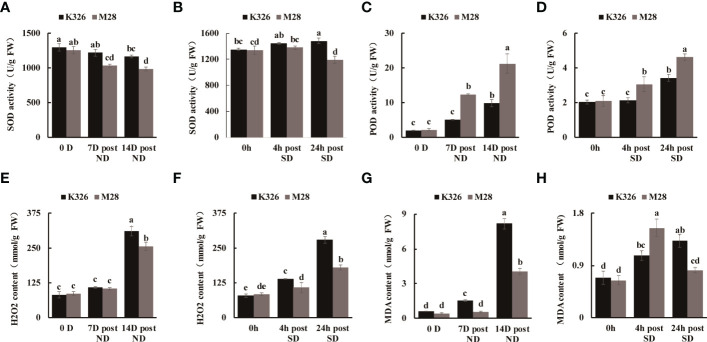
Biochemical comparisons between tobacco K326 and M28 at different time points after exposing to ND and SD, respectively. **(A, B)** SOD activity at 0 D,7 D,14 D post ND, and 0h,4h,24h between tobacco K326 and M28. **(C, D)** POD activity at 0 D,7 D,14 D post ND, and 0h,4h,24h between tobacco K326 and M28. **(E, F)** H2O2 content at 0 D,7 D,14 D post ND, and 0h,4h,24h between tobacco K326 and M28. **(G, H)** MDA content at 0 D,7 D,14 D post ND, and 0h,4h,24h between tobacco K326 and M28. Different letters represent significant differences (P< 0.05, one-way ANOVA).The difference is not significant if there is a letter with the same marker, and significant if there is a letter with a different marker.

**Figure 2:**
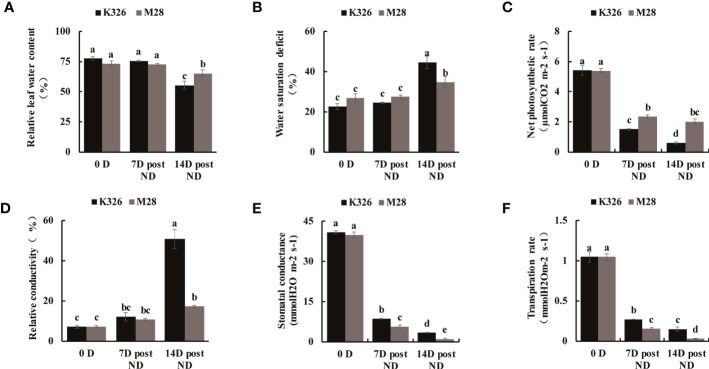
Physiological comparisons between tobacco K326 and M28 at different time points after exposing to ND. **(A, B)** Relative leaf water content at 0 D,7 D,14 D post ND, and 0h,4h,24h between tobacco K326 and M28. **(C)** Net photosynthetic rate at 0 D,7 D,14 D post ND between tobacco K326 and M28. **(D)** Relative conductivity at 0 D,7 D,14 D post ND between tobacco K326 and M28. **(E)** Stomatal conductance at 0 D,7 D,14 D post ND between tobacco K326 and M28. **(F)** Transpiration rate at 0 D,7 D,14 D post ND between tobacco K326 and M28.Different letters represent significant differences (P< 0.05, one-way ANOVA).The difference is not significant if there is a letter with the same marker, and significant if there is a letter with a different marker.

We then measured the physiological and biochemical changes between K326 and M28 at different time points after exposure to ND or SD. As compared to K326 under ND or SD treatment, M28 exhibited slightly decreased SOD activity, but markedly increased POD activity (**Figures 1A–D**). As expected, H_2_O_2_ accumulation at 14 D post ND, and 4h/24h post SD was less in M28 than in K326 (**Figures 1E, F**). As compared to K326, M28 had less content of malondialdehyde (MDA) at 7D/14D post ND and 24h post SD, but higher MDA content at 4h post SD (**Figures 1G, H**). Moreover, higher relative leaf water content at 14 D post ND, higher water saturation deficit (WSD) before and after ND, higher net photosynthetic rate at 7D/14D post ND were detected in M28 than in K326 (**Figures 2A–C**). In addition, less relative conductivity, stomatal conductance and transpiration rate at 7D/14D post ND were detected in M28 as compared to K326 (**Figures 2D–F**). These result indicated that drought stress caused less damage in M28 than in K326.

Taken together, all above analyses show that differential morphological, physiological and biochemical changes occur between K326 and M28 during drought stress, confirming that M28 is more resistant to drought stress than K326.

### Differential gene expression in response to drought treatment between K326 and M28

To assess if genes are differentially expressed between K326 and M28 during drought stress, we conducted RNA-seq using K326 and M28 under drought treatment at multiple time points (0, 1, 2, 4 and 8 h), and obtained well-correlated biological triplicates for each time point (**Supplementary Figure S3**). After identifying time-point-related differentially expressed genes (DEGs, padj < 0.01 & $\left|{\text{log}}_{2}^{\text{FoldChange}}\right|$ > 1) in K326/M28 or between K326 and M28 before and post drought treatment (**Supplementary Table S1**), we found that 700/782 genes were down- and up-regulated, respectively, in M28 relative to K326 before drought treatment. These DEGs are most likely caused by EMS-induced genomic sequence variations between two samples. For post drought treatment at the time point for each sample or between samples as indicated (**Supplementary Table S1**), we found that the highest number of DEGs occurred at 8 h post drought treatment, indicative of time point-dependent gene expression changes in response to drought treatment.

**Figure 3:**
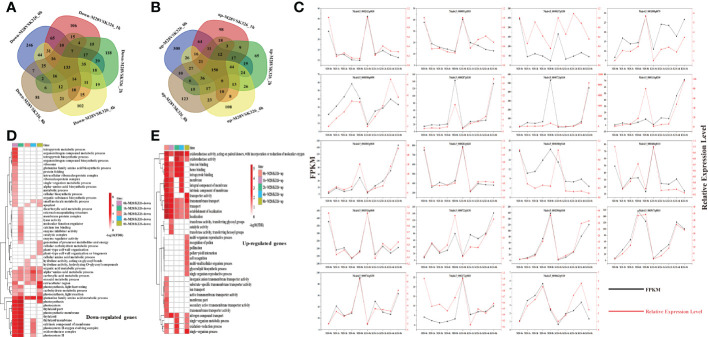
Characterization of differentially expressed genes before and after drought treatment in M28 or K326. **(A)** Pairwise comparisons of down-regulated genes at the same time point between M28 and K326 after drought treatment. **(B)** Pairwise comparisons of up-regulated genes at the same time point between M28 and K326 after drought treatment. **(C)** 19 DEGs were randomly selected for RT-qPCR assay. The red line represents the relative expression, and the black line represents the FPKM values. **(D)** GO term enrichment analyses of down-regulated genes at the same time point between M28 and K326 after drought treatment. **(E)** GO term enrichment analyses of up-regulated genes at the same time point between M28 and K326 after drought treatment.

After pair-wise comparisons of DEGs between K326 and M28 at each individual time point, we found that 133 down-regulated genes and 150 up-regulated genes were shared for all time points (**Figures 3A, B**). Besides, we observed that some genes exhibited time-point-dependent expression (**Figures 3A, B**). For instance, 246/300 and 81/123 genes were down/up-regulated at 0 and 8 h, respectively. Among 1,327 up-regulated and 1,249 down-regulated genes occurred in M28 relative to K326, we found that 545 and 549 genes corresponding to the drought-induced up- and down-regulation, respectively, in M28, by contrast, 482 up-regulated and 454 down-regulated genes were possibly caused by genomic sequence variations in combination with drought treatment (**Supplementary Figure S4**). We randomly chose 19 DEGs for RT-qPCR assay, and observed a similar expression trend as RNA-seq data (**Figure 3C**), confirming the reliability of DEGs identified by analyzing RNA-seq data.

To examine if these DEGs have any specific biological functions facilitating drought tolerance, we conducted GO terms enrichment assays. We found that 133 common down-regulated genes were overrepresented in metabolic processes such as oxoacid and organic acids (**Supplementary Figure S5**); distinct GO terms occurred in time-point-related DEGs (**Figures 3D, E**). For example, down-regulated genes at 1 h post drought treatment were more enriched in dicarboxylic acid metabolic processes, regulator activities and calcium ion binding; by contrast, up-regulated genes at 8 h post drought treatment were more enriched in reproductive events, such as pollination, pollen-pistil interaction, recognition of pollen and single/multi-organism reproductive processes.

Similarly, we conducted pair-wise comparisons and GO terms enrichment of time-point-related DEGs in M28 or K326 (**Supplementary Figures S6A, B**). We found that differential GO terms mainly occurred in up-regulated genes between M28 and K326 (**Supplementary Figure S6C**). As illustrated in **Figure 3D**, up-regulated genes in M28 had more enriched terms in response to heat or temperature stimulus, by contrast, up-regulated genes in K326 were more enriched in GO terms associated with the regulation of cellular and biological processes, gene transcription and regulation of RNA biosynthetic or metabolic processes.

Collectively, these results indicate that drought induced DEGs between M28 and K326 had distinct GO terms, which may partially explain differential drought responses between two materials.

### Differential metabolic changes in response to drought treatment between M28 and K326

To determine if drought treatment causes differential accumulation of metabolites between M28 and K326, we generated time-point-related metabolomics data *via* LC-MS in M28 and K326 before and after drought treatment. Principal component analysis (PCA) showed a clear separation between M28 and K326. We noticed that, after drought treatment, M28 mainly exhibited the first principal component (PC1) changes, whereas K326 mainly exhibited the second principal component (PC2) changes (**Supplementary Figure S7**), indicating dramatic changes of metabolic profiles between M28 and K326 before and after drought treatment.

**Figure 4:**
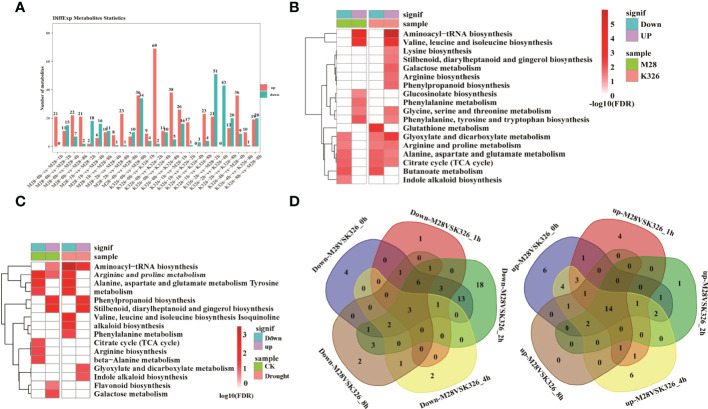
Differential metabolic changes in response to drought treatment between M28 and K326. **(A)** Histogram showing the number of differential metabolic changes in response to time-point-related drought-induced in M28 or K326 or between M28 and K326. **(B)** KEGG pathway enrichment analyses of up- and down-regulated metabolites in K326 or M28 under drought treatment. **(C)** KEGG pathway enrichment analyses of metabolites that were up- or down-regulated before (CK) or under drought treatment. **(D)** Pairwise comparisons of down- (left) and up-regulated (right) metabolites at the same time point between M28 and K326 after drought treatment.

We then identified drought induced metabolites (DiffExp, a variable importance in projection (VIP) score >1, an FC > 1.5 or <0.66 and *p* < 0.05) in M28 or K326, and between M28 and K326 using orthogonal partial least squares discriminant analysis (OPLS-DA) (**Supplementary Table S2**). As shown in **Figure 4A**, the highest number (69) of up-regulated DiffExp metabolites was observed at 2 h post drought treatment in K326, and the highest number (51) of down-regulated DiffExp metabolites was observed at 2 h post drought treatment between K326 and M28. To assess if DiffExp metabolites are involved in any metabolic pathways, we conducted KEGG pathway analyses using DiffExp metabolites between K326 and M28 (**Figure 4B**). In addition to some metabolic pathways related to up or down-regulated DiffExp metabolites shared between K326 and M28, distinct metabolic pathways occurred between K326 and M28. For example, down-regulated DiffExp metabolites were more enriched in indole alkaloid biosynthesis in M28, whereas were more enriched in glutathione metabolism in K326; up-regulated DiffExp metabolites were overrepresented in glucosinolate biosynthesis and phenylalanine metabolism in M28, whereas were more enriched in lysine/arginine/gingerol/phenylpropanoid biosynthesis, galactose metabolism in K326.

We also conducted KEGG pathway analyses using DiffExp metabolites before and after drought treatment, including 8/13 up-regulated and 4/28 down-regulated metabolites corresponding to CK and post drought treatment, respectively (**Supplementary Figure S8**). Differential enrichment of metabolic pathways was observed between CK- and drought-related DiffExp metabolites (**Figure 4C**). For instance, drought-related up-regulated metabolites were overrepresented in glyoxylate and dicarboxylate metabolism and indole alkaloid biosynthesis; by contrast, CK-related up-regulated metabolites were overrepresented in galactose metabolism and flavonoid biosynthesis. After pairwise comparisons, we found that there were 3 down-regulated and 14 up-regulated metabolites present at all time points in M28 post drought treatment (**Figure 4D**). According to KEGG pathway analyses, we found that 2 down-regulated metabolites (spermidine and putrescine) were involved in arginine/proline/glutathione metabolisms (**Supplementary Figure S9**); 3 up-regulated metabolites (gluconic acid, 5-O-p-coumaroylquinic acid and chlorogenic acid) were involved in pentose phosphate pathway and stilbenoid diarylheptanoid and gingerol biosynthesis (**Supplementary Figure S10**).

Furthermore, we conducted KEGG enrichment analysis using all differentially expressed metabolites significantly changed post drought treatment. We observed and 29 significantly enriched KEGG pathways (**Supplementary Figure S11**), including Nitrogen_/Alanine_/Arginine_/Pyruvate_metabolism pathways (**Supplementary Figure S12**) involved in drought stress (Winter and Holtum, 2015; Shen et al., 2017; Cui et al., 2019; Parthasarathy et al., 2019).

Collectively, these results indicate that drought induced DiffExp metabolites between M28 and K326 had distinct GO terms, possibly leading to differential drought responses between two materials.

### Integrated analyses of transcriptome and metabolome related to drought stress

**Figure 5:**
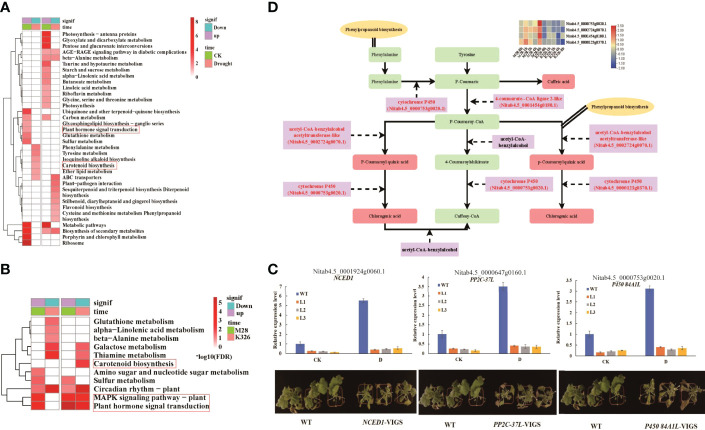
Integrated analyses of transcriptome and metabolome. **(A)** KEGG pathway enrichment analyses of genes that were up- or down-regulated before (CK) or under drought treatment. **(B)** KEGG pathway enrichment analyses of up- and down-regulated genes in K326 or M28 under drought treatment. **(C)** Relative expression levels (up) and phenotypes (down) of VIGS-mediated knock-down of gene *NCED1*, *PP2C-37L* and *P450 84A1L* before or post drought treatment. **(D)** Overview of DEGs and metabolites involved in “Phenylpropanoid biosynthesis” and “Flavonoid biosynthesis”. The red representing differentially expressed genes and differential metabolites that are significantly enriched in the pathways of Phenylpropanoid biosynthesis terms and Flavonoid biosynthesis pathway. Four differentially expressed genes with relative expression levels (FPKM value) in the M28 and K326 are shown in the heatmap.

To investigate if DEGs directly result in DiffExp metabolites during drought treatment, we first conducted KEGG pathway analyses using drought inducible DEGs between M28 and K326. We observed differential KEGG pathways occurred in down- and up-regulated genes between M28 and K326 before and after drought treatment (**Figure 5A**). For example, before drought treatment, down-regulated genes were mainly enriched in pathways associated with biosynthesis of secondary metabolites and ubiquinone, ribosome and porphyrin and chlorophyll metabolism; by contrast, up-regulated genes were overrepresented in pathways related to phenylalanine/tyrosine metabolism, ABC transporters and carotenoid biosynthesis. Particularly, we observed that down-regulated genes (**Supplementary Table S3**) were involved in plant hormone signal transduction such as auxin-responsive or induced proteins and ethylene-response factors, while up-regulated genes (**Supplementary Table S4**) were involved in carotenoid biosynthesis such as abscisic acid 8’-hydroxylase and 9-cis-epoxycarotenoid dioxygenase NCED1 for biosynthesis of ABA (Song et al., 2021). After drought treatment, down-regulated genes were mainly enriched in pathways associated with photosynthesis, carbon fixation, glutathione/alpha-linolenic acid/galactose/arginine and proline metabolism, biosynthesis of unsaturated fatty acids and amino acids; by contrast, up-regulated genes were overrepresented in pathways related to glucerolipid/C5-branched dibasic acid metabolism, and flavonoid/phenylpropanoid/brassinosteroid/zeatin biosynthesis.

Similarly, we conducted KEGG pathway analyses using drought inducible DEGs in M28 or K326 (**Figure 5B**). We observed differences in up- and down-regulated genes in M28 or K326. We found that distinct pathways occurred in up- and down-regulated genes between M28 and K326. For example, compared with genes in K326, down-regulated genes in M28 were more enriched in amino sugar and nucleotide sugar metabolism; up-regulated genes in M28 were more enriched in glutathione/alpha-linolenic acid/beta-alanine metabolism, up-regulated genes in K326 were more enriched in carotenoid biosynthesis. In particular, we found that up-regulated genes in K326 (**Supplementary Table S5**) were involved in carotenoid biosynthesis, down-regulated genes in M28 and DEGs in K326 (**Supplementary Table S6**) were involved in MAPK signaling pathway like PP2C and plant hormone signal transduction. These results indicated that more enriched KEGG pathways may facilitate more drought tolerance in M28 than K326. To further confirm involvement of genes *NCED1*, *PP2C-37L* and *P450 84A1L* in drought responses, we conducted VIGS-mediated knock-down of each individual genes in combination with drought treatment. We found that the sensitivity of each VIGS line to drought treatment was directly associated with the expression level of the corresponding gene (**Figure 5C**), indicative of positive roles of each gene in drought responses in tobacco.

After associating DEGs- and DiffExp metabolites-related KEGG pathways, we detected 16 pathways shared between DEGs and DiffExp metabolites (**Supplementary Table S7**), such as phenylalanine, galactose and glutathione metabolisms. We noticed that DEGs were markedly enriched in “phenylpropanoid biosynthesis” terms and “flavonoid biosynthesis” pathways. We then combined our RNA-seq and metabolomics data to construct a gene-metabolite network (**Figure 5D**). We observed that cytochrome P450 gene (*Nitab4.5_0000753g0020.1*) was involved in several key steps for phenylpropanoid biosynthesis such as cinnamic acid to p-coumaric acid, and p-coumaroyl quinic acid to chlorogenic acid, indicating that this gene plays important roles in controlling phenylpropanoid biosynthesis in tobacco.

**Figure 6:**
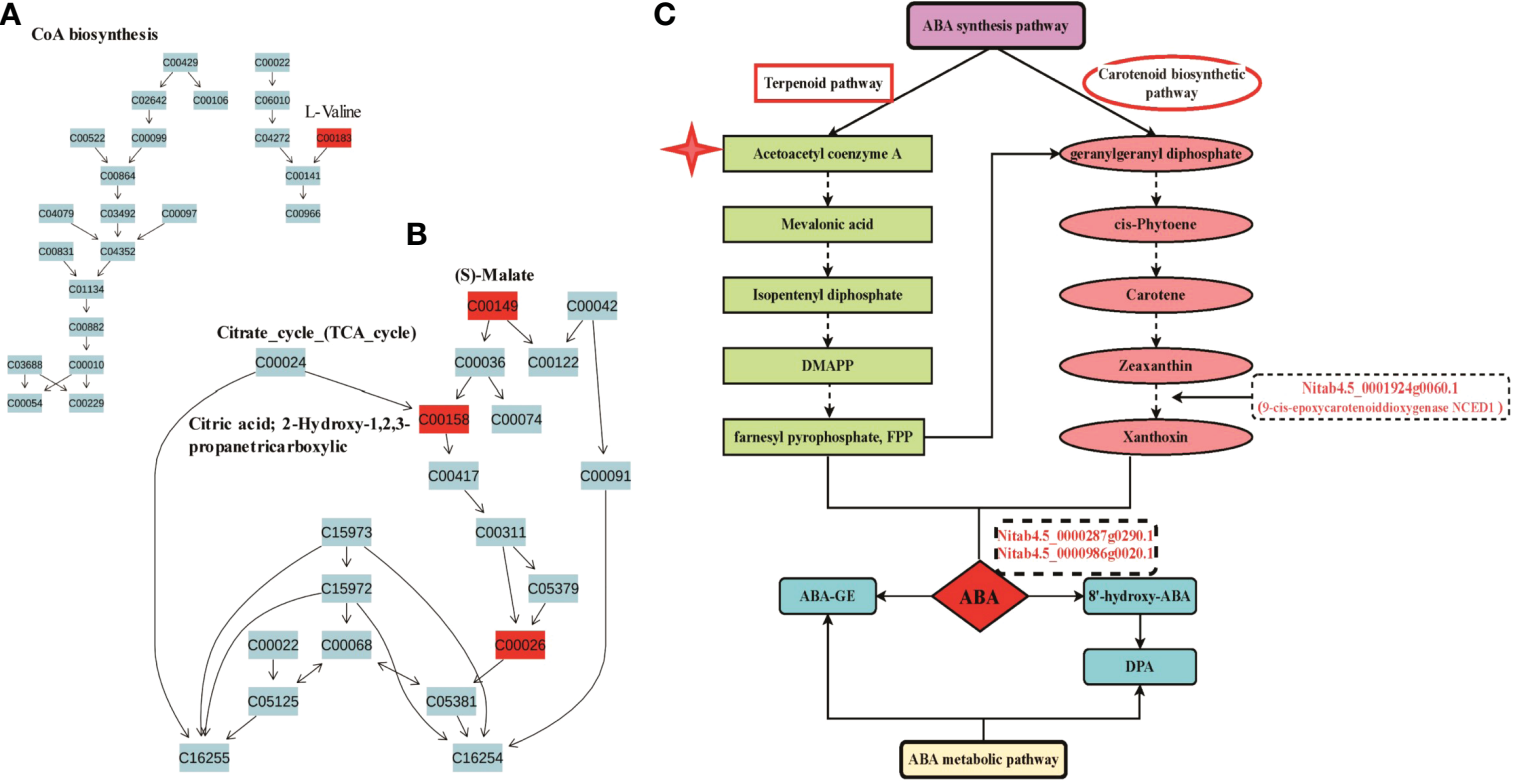
Involvement of Abscisic acid in drought responses in tobacco. **(A)** Overview of the KEGG pathway of CoA biosynthesis. The red indicating significantly up-regulated metabolites between M28 and K326 after drought treatment. **(B)** Overview of the KEGG pathway of Citrate cycle (TCA cycle). The red indicating significantly up-regulated metabolites between M28 and K326 after drought treatment. **(C)** Involvement of DEGs in combination with metabolites in synthesis and decomposition pathways of Abscisic acid in tobacco.

In addition, we found that L-Valine (C00183) was involved in Acetoacetyl coenzyme A biosynthesis, which is the key component in terpenoid pathways for synthesis of ABA (Boba et al., 2020) (**Figure 6A**); S-Malate(C00149), citric acid (C00158) and 2-Oxoglutarate (C00026) were involved in Citrate-cycle (TCA-cycle) (**Figure 6B**), leading to production of Acetoacetyl coenzyme A for ABA synthesis. Among DEGs, we found that NCEDs is the key enzyme catalyzing Zeaxanthin to form Xanthoxin during ABA synthesis pathway, and *Nitab4.5_0000287g0290.1* and *Nitab4.5_0000986g0020.1* are key enzymes catalyzing ABA to form 8’-hydroxy-ABA during ABA metabolic pathway (**Figure 6C**). Besides, we found that four DEGs, such as *Nitab4.5_0002255g0010.1*, *Nitab4.5_0001650g0160.1* (*glutamine synthetase*), *Nitab4.5_0000777g0010.1* (*glutamine synthetase*) and *glutamate synthase* (*GOGAT*), were involved in nitrogen metabolism, leading to synthesis of glutamate (Glu) starting from ${\text{NO}}_{3}^{-}$ / ${\text{NO}}_{2}^{-}$ (**Supplementary Figure S13**). Glutamate has been reported to involve in plant growth and development, and stress response and adaptation in plants (Qiu et al., 2019). We validated expression of *Nitab4.5_0001454g0180.1* and *Nitab4.5_0000123g0370.1* by using RT-qPCR assay, which were identified from metabolic pathways linked with RNA-seq. Both genes were more expressed at 1 h, 2 h and 4 h but less at 8 h in M28 as compared to K326 post drought stress (**Supplementary Figure S14**).

Collectively, all above analyses indicate that a subset of drought induced DEGs function in drought response through the regulation of DiffExp metabolite synthesis in tobacco.

### Co-expression network in relation to drought responses

**Figure 7:**
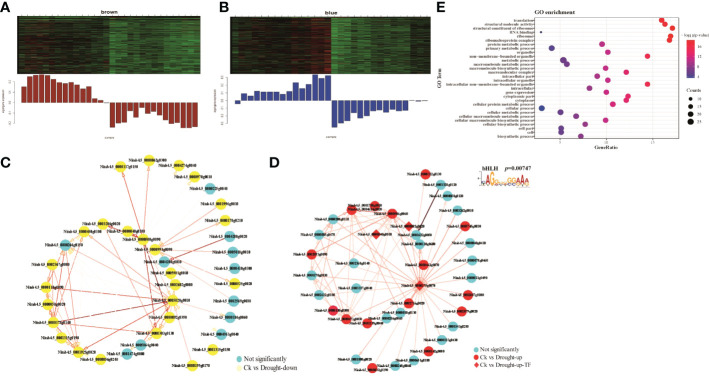
WGCNA analyses. **(A)** Module gene expression pattern in the brown module, red representing up-regulated genes, the green representing down-regulated genes. **(B)** The correlation network of genes in the brown module. A gene network is constructed by WGCNA, in which each node represents a gene, and the connecting line between genes represents the co-expression correlation, the blue representing not significant genes and the yellow representing down-regulated genes between M28 and K326 before and after drought treatment. The genes with weights >0.1 are visualized by Cytoscape. **(C)** The correlation network of genes in the blue module. **(D)** The correlation network of genes in the blue module. The red representing up-regulated genes and the blue representing not significant genes. The diamond representing the TF in the co-expression network. **(E)** GO term enrichment analyses of down-regulated genes in the brown module.

To interrogate if functions of transcription factors (TFs) in drought responses are mediated by their interactive genes, we performed Weighted Gene Co-Expression Network Analysis (WGCNA) with 2,568 DEGs. We obtained 13 modules containing co-expression genes according to the WGCNA package function (**Supplementary Figure S15**). We found that blue/brown and MEtan models displaying a high correlation with up- and down-regulated genes, respectively, in M28 at 8h post drought treatment (**Figures 7A, B**; **Supplementary Figure S16A**). There were 26 up-regulated and 24 down-regulated genes in brown and Tan modules, respectively (**Supplementary Table S8**). Blue module contained 15 up-regulated genes and 3 TFs (*Nitab4.5_0000175g0070*, *bHLH*; *Nitab4.5_0000540g0010*, *C3H*; *Nitab4.5_0000082g0020*, *CCAAT*) (**Supplementary Table S9**). We then constructed TF-centered co-expression networks for those 3 modules (**Figures 7C, D**; **Supplementary Figure S16**). In blue module, we observed interactions between *bHLH* and some of up-regulated genes (**Figure 7D**). To confirm this possibility, we conducted *de novo* motif identification using promoter regions of all up-regulated genes in blue module. We indeed detected a typical CAGGGGGGAAA motif for the potential binding of bHLH, which is similar to CACGTG motif associated with bHLH69 identified through DAP-seq data in *Arabidopsis* (O'Malley et al., 2016).

**Figure 8:**
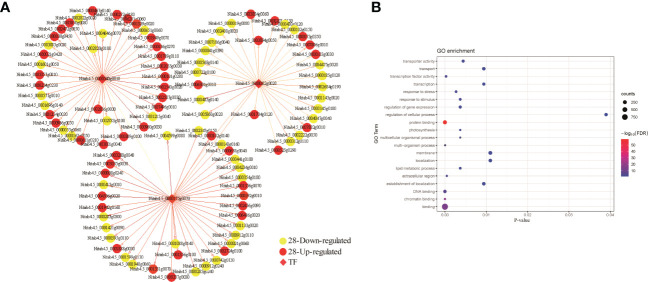
Prediction of TF regulatory networks using the co-expression correlation network of three TFs in the blue module. **(A)** The correlation network of genes related to regulation of the three TFs. The red representing up-regulated genes and the yellow representing down-regulated genes between M28 and K326 before and after drought treatment. The direction of the arrow indicates the regulatory relationship. The genes with weights > 0.1 are visualized by Cytoscape. **(B)** GO term enrichment analyses of down- and up-regulated genes related to regulation of the three TFs.

To examine if genes in each module mentioned above have any biological implications, we conducted GO term enrichment assays. We found that genes in blue module were significantly enriched in functions associated with protein translation-related functions, structural molecule activity, gene expression and cytoplasm (**Figure 7E**). By contrast, no significant GO terms were detected for genes in brown and tan modules. To interrogate how *bHLH* is involved in drought responses in M28, we constructed *bHLH*-related regulatory network and found that *bHLH* can directly interact with *CCAAT* TF, and may indirectly interact with C3H (**Figure 8A**). We further found that CCAAT and C3H TFs can interact with 10 and 25 up-regulated genes in M28 during drought treatment. After performing GO enrichment analyses, we found that C3H-regulated genes were mainly involved in nucleoside-triphosphatase activity, pyrophosphatase/hydrolase activity, acting on acid anhydrides, in phosphorus-containing anhydrides; by contrast, CCAAT regulated genes exhibited functions highly enriched in methyltransferase activity, nucleotide binding and stress responses (**Figure 8B**). Thus, these analyses provided evidence showing direct and indirect involvement of *bHLH* TF in drought responses in tobacco.

Collectively, all above analyses indicate that, in addition to direct functions of a subset of TFs in drought stress, they can be indirectly involved in drought responses through the regulatory networks in tobacco.

## Discussion

To acclimate various severe stress factors for normal development or survival, sessile plants have to adjust multifaceted changes, including physiological, biochemical and metabolic changes, reprogramming of gene expression and DNA methylation, chromatin modification and ncRNA-related epigenetic changes as well (Covarrubias and Reyes, 2010; Kim et al., 2010; Ramakrishna and Ravishankar, 2011; Krasensky and Jonak, 2012; Kumar, 2014; Kim et al., 2015; Janiak et al., 2016; Takahashi et al., 2018; Liu and He, 2020; Bhadouriya et al., 2021; Hussain et al., 2021; Kumar et al., 2021). In this study, we explored possible mechanisms underlying contrasting drought responses between K326 and its derived mutant M28, including physiological and biochemical, transcriptional and metabolic levels.

Resistance of M28 to drought can be achieved at physiological and biochemical levels. M28 exhibited less H2O2 and MDA accumulation and higher POD activities as compared to K326 post drought treatment, particularly for a long duration of ND (14D) or SD (24h). Moreover, M28 had higher photosynthetic rate, less stomatal conductance and transpiration rate. These changes lead to less membrane damage and water loss and higher capacity for producing energy through photosynthesis, facilitating M28 to adapt to drought stress through alleviating drought related damage. Similar findings have been reported in tobacco or other plant species in response to drought or other stress factors such as salt, cold and heat (Su et al., 2017; Jamshidi Goharrizi et al., 2020; Mude et al., 2020).

At the transcription levels, down-regulated genes in M28 were mainly involved in some fundamental biological processes, including cellular and biological processes, gene transcription and the regulation of RNA biosynthetic or metabolic processes, therefore plants with the down-regulation of these genes can save more energy to cope with drought stress for better survival; while up-regulated genes in M28 had functions overrepresented in heat or temperature stimuli (**Figure 3D**) or reproductive events, such as pollination, pollen-pistil interaction, recognition of pollen and single/multi-organism reproductive processes (**Figures 3D, E**). Those up-regulated genes either help the plants alleviate impairment of drought stress or promote transition from the vegetative stage to the reproductive stage, leading to rapidly complete the whole lifecycle and produce seeds, and facilitating better development and survival of plants under drought conditions. For instance, *NtEXGT* gene, encoding xyloglucan endotransglucosylases/hydrolases (XTHs) in *Nicotiana tabacum* L., has been reported to be involved in abiotic stress response through ABA-dependent signaling pathway (Kuluev et al., 2017). It was slightly induced by drought stress in *Nicotiana tabacum* L. (Kuluev et al., 2017), by contrast, we found that it was down-regulated in K326 but almost no changes in M28 in different time points after drought treatment. The contrasting change in expression of this gene is possibly caused by variations in cis-regulatory elements.

Furthermore, metabolic analyses provided evidence showing that enhanced resistance of M28 to drought stress was ABA signaling pathway-dependent. We detected that Diffexp metabolites were involved in valine biosynthesis, citrate cycle (TCA cycle), carotenoid biosynthesis KEGG pathway (**Figures 4C, B**). Those secondary metabolites are necessary for ABA biosynthesis (Xie et al., 2022) (**Figure 6**). The plant hormone abscisic acid (ABA) is the key active regulator functioning in plant stress response and tolerance through the regulation of stress-responsive genes or stomatal movement and modulating ROS homeostasis (Smyk-Randall and Brown, 1987; Fujita et al., 2011; Nakashima and Yamaguchi-Shinozaki, 2013; Dong et al., 2021). Transcriptome and metabolome integrative analyses linked DEGs with the Diffexp metabolites. For instance, drought induced NCEDs, the key enzyme catalyzing Zeaxanthin to form Xanthoxin during ABA synthesis pathway, and *Nitab4.5_0000287g0290.1* and *Nitab4.5_0000986g0020.1*, key enzymes catalyzing ABA to form 8’-hydroxy-ABA during ABA metabolic pathway, mitigated impairment of drought stress through affecting ABA biosynthesis and metabolic pathway (**Figure 6C**). In addition, Nitrogen_/Alanine_/Arginine_/Pyruvate_metabolism pathways involved in drought responses were more enriched in M28 than K326. Pyruvate has been found to promote stomatal closure through inducing ROS production in response to drought stress, thus regulating stomatal motility to reduce transpiration rate and avoid water loss (Shen et al., 2017). Alanine accumulation acts as a generic stress response molecule to help mitigate multiple stress factor induce damages including drought, thereby facilitating plants to survive during stress responses (Parthasarathy et al., 2019). Arginine serves as precursor for nitric oxide (NO) and polyamines, thereby involving in biotic and abiotic responses in plants (Winter et al., 2015).

TFs are key regulators, acting individually or through regulatory networks, responsible for adaptive responses to abiotic stresses (Golldack et al., 2014; Manna et al., 2021). *MYB* TFs, which can be induced by drought stress, play vital roles in plants responding to drought stress, including maintaining cellular or organ structures and functions, adjusting stomatal movement and the regulation of secondary metabolisms (Dubos et al., 2010; Shin et al., 2011; Li et al., 2019a). *TabHLH1* was found to function in drought and salt stress through regulating expression of genes involving in ABA signaling pathway (Yang et al., 2016). Overexpression of *TaSNAC8-6A*, a drought responsive gene, and *TaABL1* can facilitate drought tolerance in transgenic *Arabidopsis* or wheat (Xu et al., 2014; Mao et al., 2020). Involvement of overexpressed *GhWRKY17* in enhancing drought tolerance is mediated by ABA signaling pathway and control of reactive oxygen species (ROS) generation in transgenic *Nicotiana benthamiana* (Yan et al., 2014). Through regulatory network analyses, we found that drought induced TFs (*Nitab4.5_0000175g007*, *bHLH*; *Nitab4.5_0000540g0010*, *C3H*; *Nitab4.5_0000082g0020*, *CCAAT*) can regulate expression of genes responsible for ABA biosynthesis (**Figure 7D**), thereby involving in drought responses in tobacco. It has been reported that *bHLH* is involved in stress-response and iron homeostasis in different plants (Wang et al., 2015; Qiu et al., 2016; Khan et al., 2018; Wang et al., 2018; Kobayashi et al., 2019).

## Conclusion

Our study provides evidence showing that drought tolerance of M28 can be achieved at multiple levels. At biochemical and physiological levels, it elevates POD activity, net photosynthesis rate along with reducing H2O2 and MDA accumulation, decrease of relative conductivity, stomatal conductance and transpiration rate; at transcriptional and metabolic levels, it exhibits differential gene expression and metabolite changes directly and indirectly involved in drought responses, thereby facilitating plants to adapt to drought stress.

## Data availability statement

The datasets presented in this study can be found in online repositories. The names of the repository/repositories and accession number(s) can be found in the article/**Supplementary Material**.

## Author contributions

WZ and RH conceived the study. ZHe analyzed the data. YL, PY, CY, RH, ZHu and JY performed the experiments. XC and QW assisted data analyses. BG edited the manuscript. ZHe and WZ wrote the manuscript with contribution from all authors. All authors contributed to the article and approved the submitted version.

## Supplementary material

Supplementary Table S3 Down-regulated gene list after drought.

Supplementary Table S4 Up-regulated gene list after drought.

Supplementary Table S5 Down-regulated gene list in K326 after drought.

Supplementary Table S6 Down-regulated gene list in M28 after drought.

Supplementary Table S7 Pathways shared between DEGs and DiffExp metabolites.
