# Supplementary material for: Transcriptomic and metabolic regulatory network characterization of drought responses in tobacco
Source: Front Plant Sci. 2023 Jan 18;13:1067076. doi: 10.3389/fpls.2022.1067076 (PMC9891310; doi:10.3389/fpls.2022.1067076)
Supplement: Supplementary file 1 [file DataSheet_1.pdf]

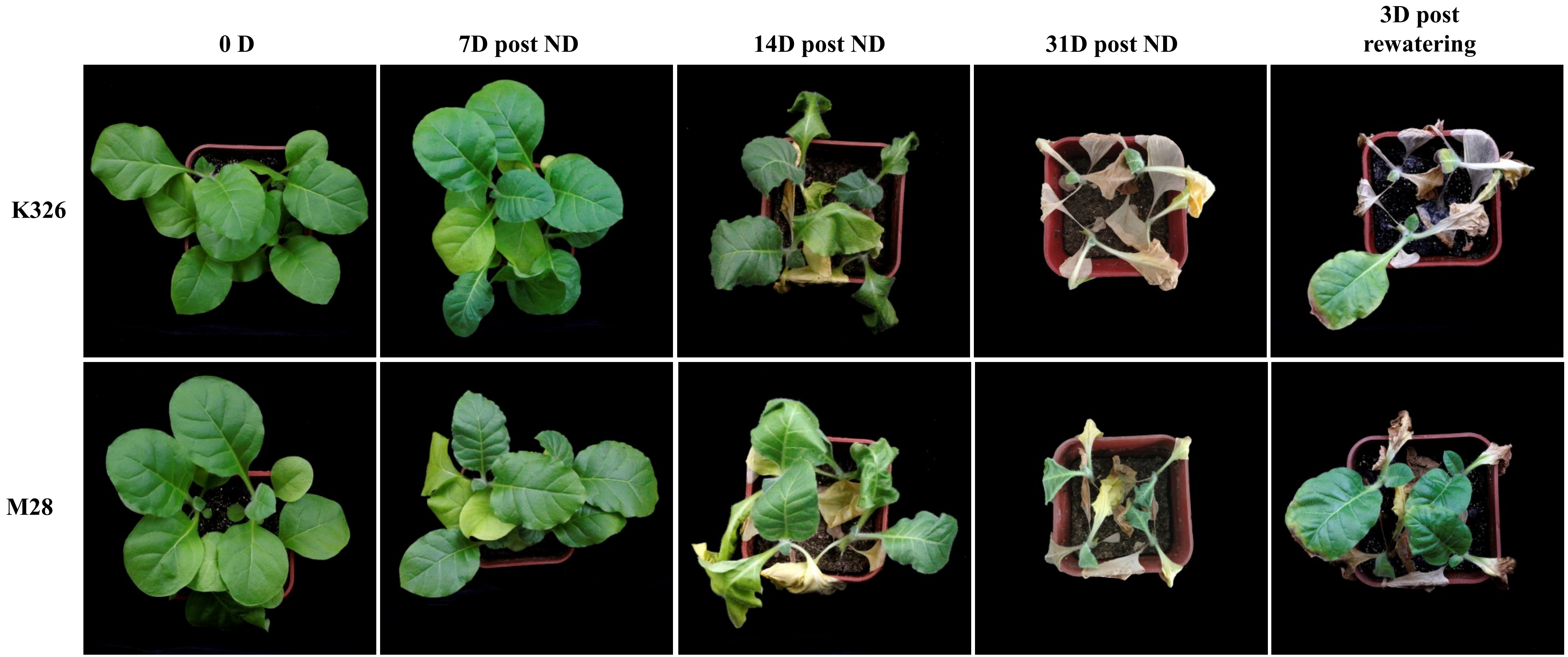


**Supplementary Figure S1 Phenotypic comparisons between tobacco K326 and M28 at different time points after exposing to natural drought (ND) stress.**

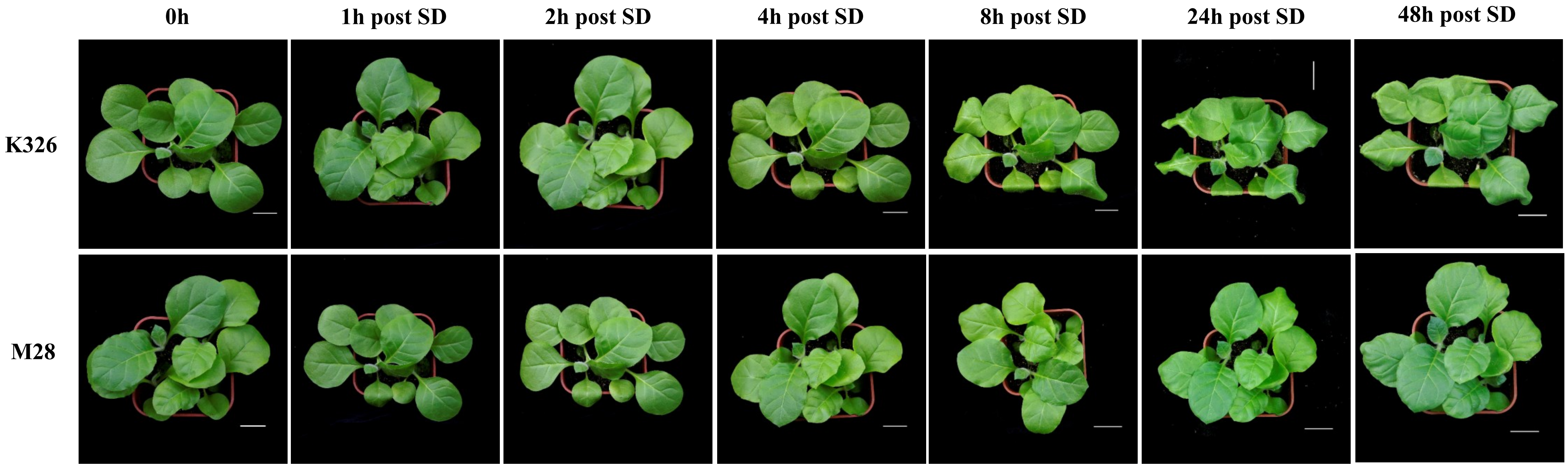


**Supplementary Figure S2 Phenotypic comparisons between tobacco K326 and M28 at different time points after exposing to 200mM mannitol treatment（simulated drought, SD).**

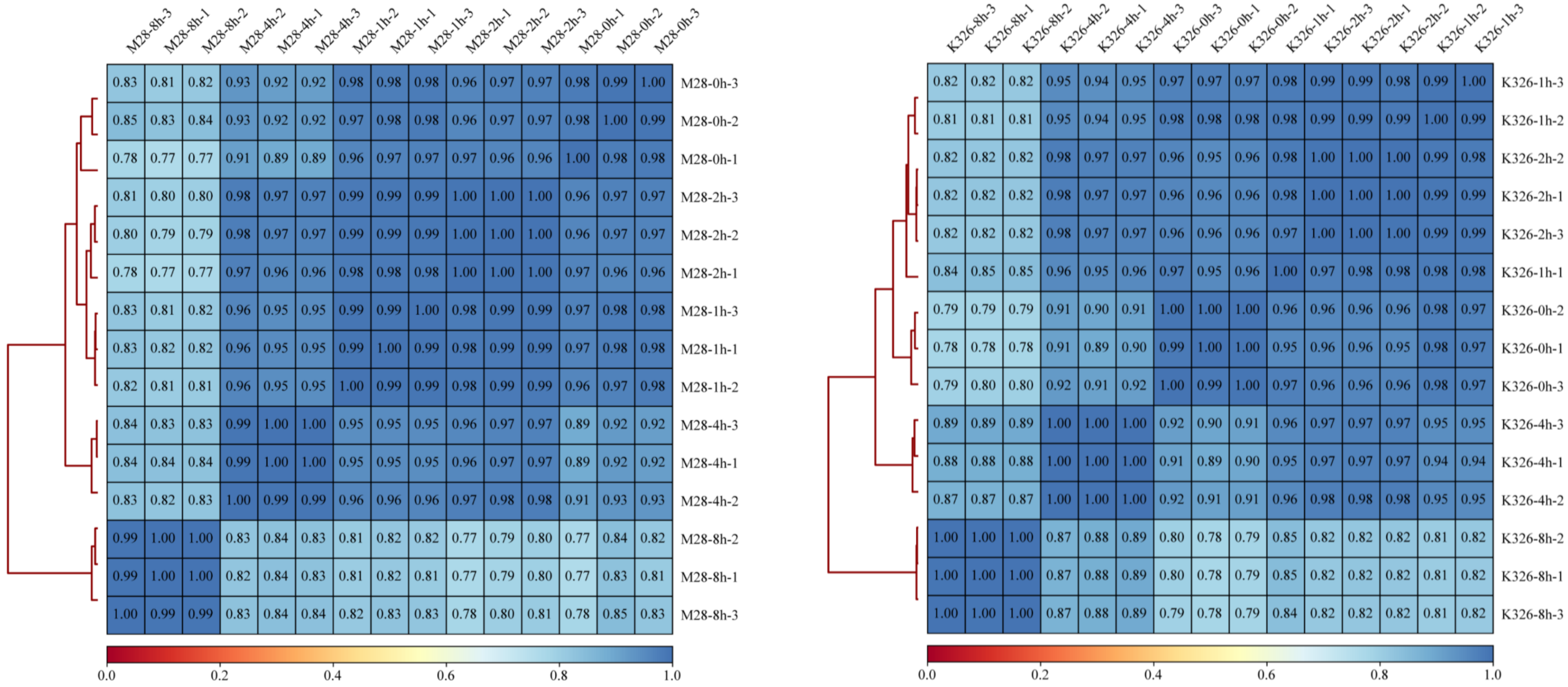


**Supplementary Figure S3 Hierarchically clustered correlation matrix of RNA-seq data generated from M28 and K326 subjected to drought treatment at different time points**. Three replicates for each time point. Pearson correlations were calculated and Hierarchically clustered by heatmap package in R.

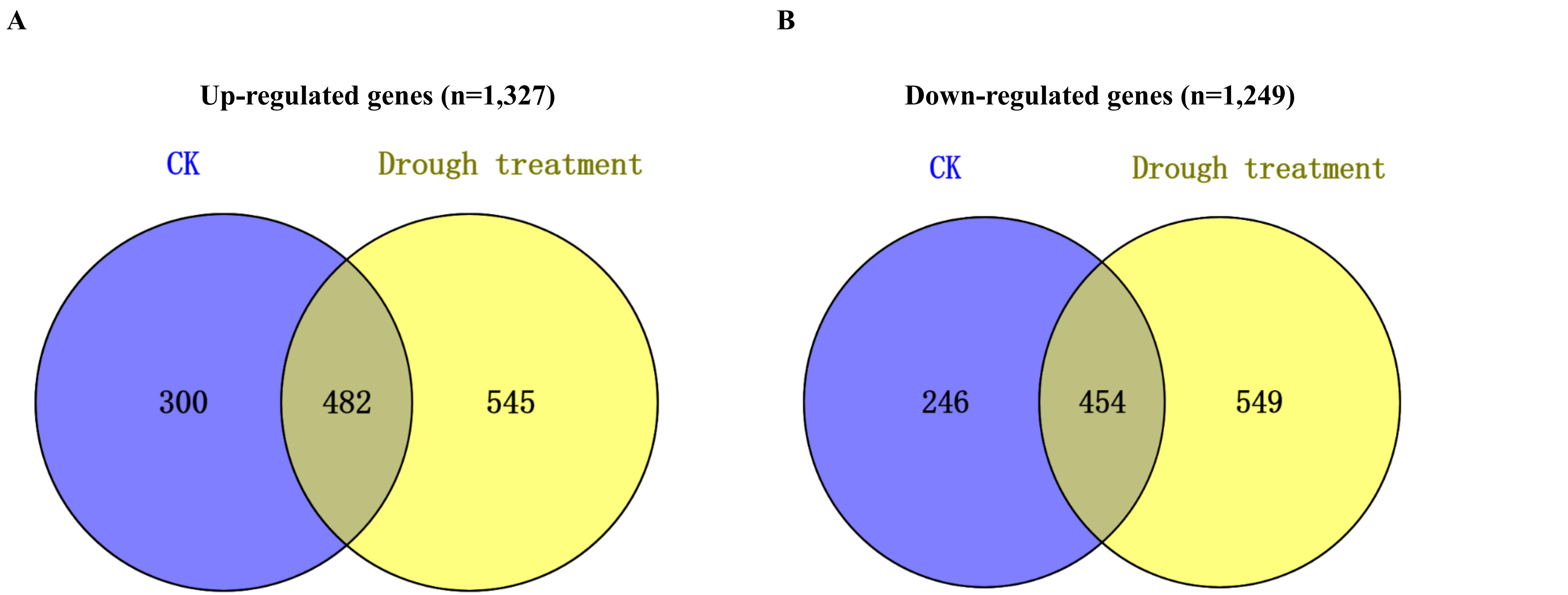


**Supplementary Figure S4 Comparisons of genes that were up- or down-regulated before (CK) or after drought treatment.**

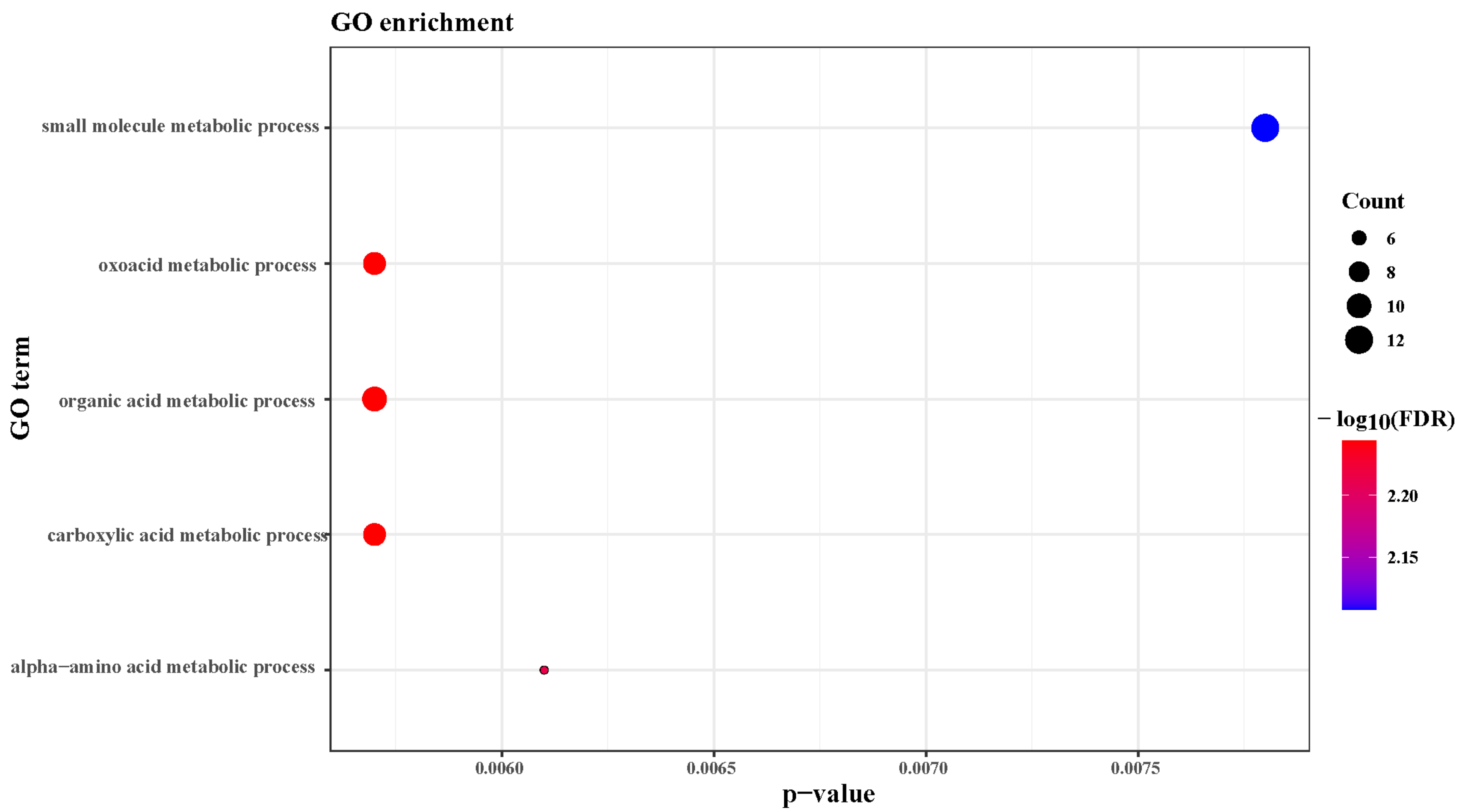


**Supplementary Figure S5 GO term enrichment analyses of common down-regulated genes for all time points under drought treatment.**

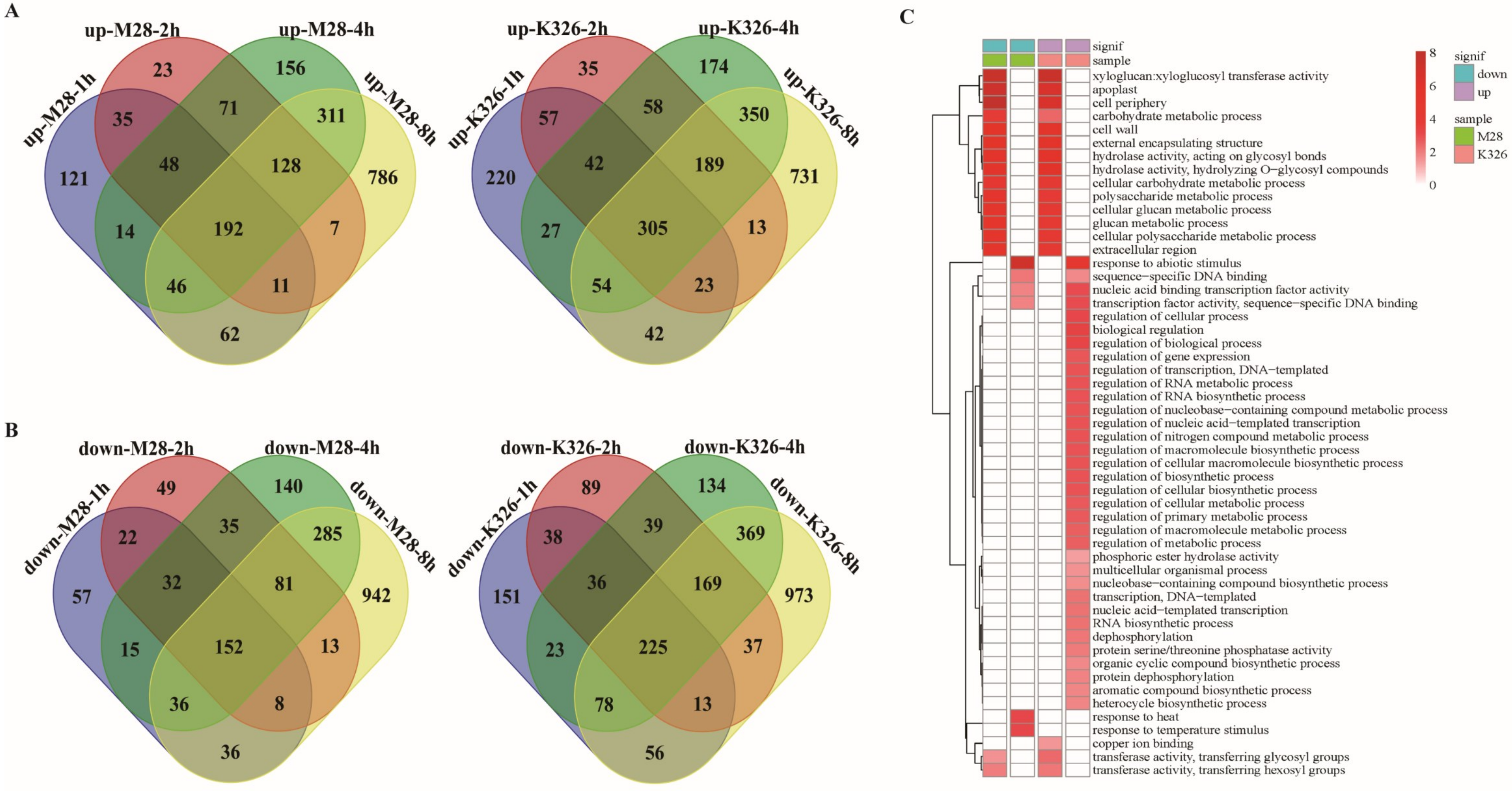


**Supplementary Figure S6 Characterization of time-point-related up- or down-regulated genes in K326 or M28 under drought treatment. (A)** Venn plots showing overlaps of time-point-related up-regulated genes in K326 (right) or M28 (left) under drought treatment. **(B)** Venn plots showing overlaps of time-point-related down-regulated genes in K326 (right) or M28 (left) under drought treatment. **(C)** GO term enrichment analyses of up- and down-regulated genes in K326 or M28 under drought treatment.

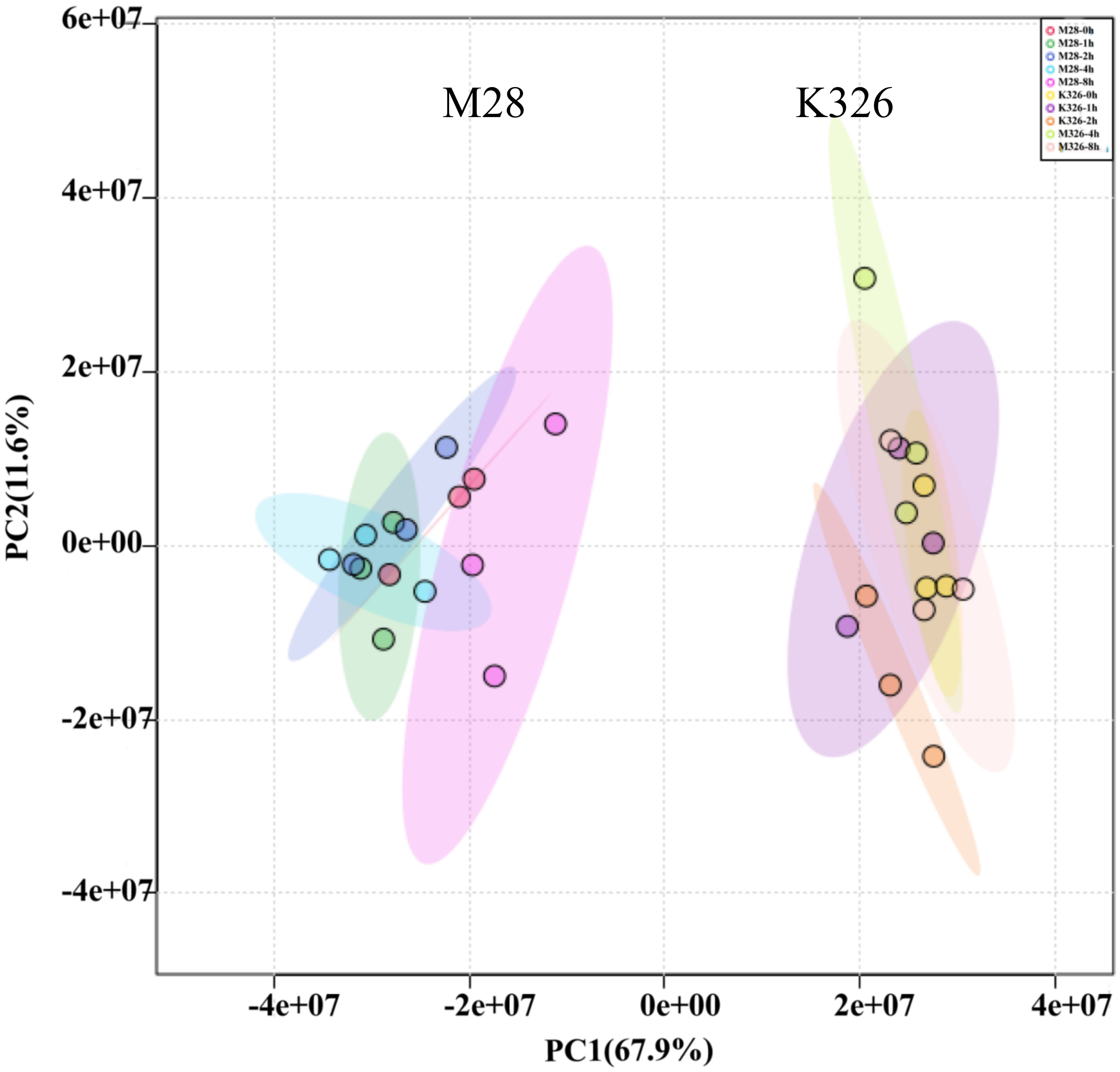


**Supplementary Figure S7 PCA of metabolome generated from M28 and K326 subjected to drought treatment at different time points.**

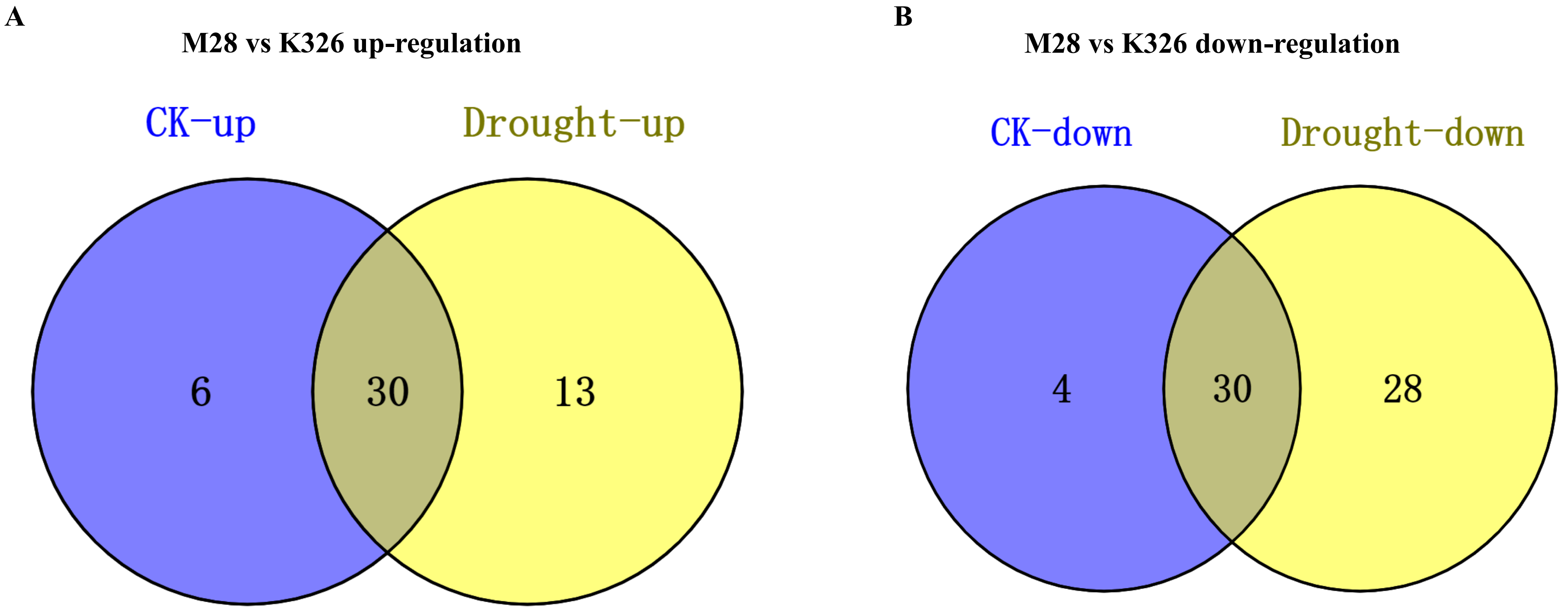


**Supplementary Figure S8 Comparisons of differential metabolites between M28 and K326 before and after drought treatment.**

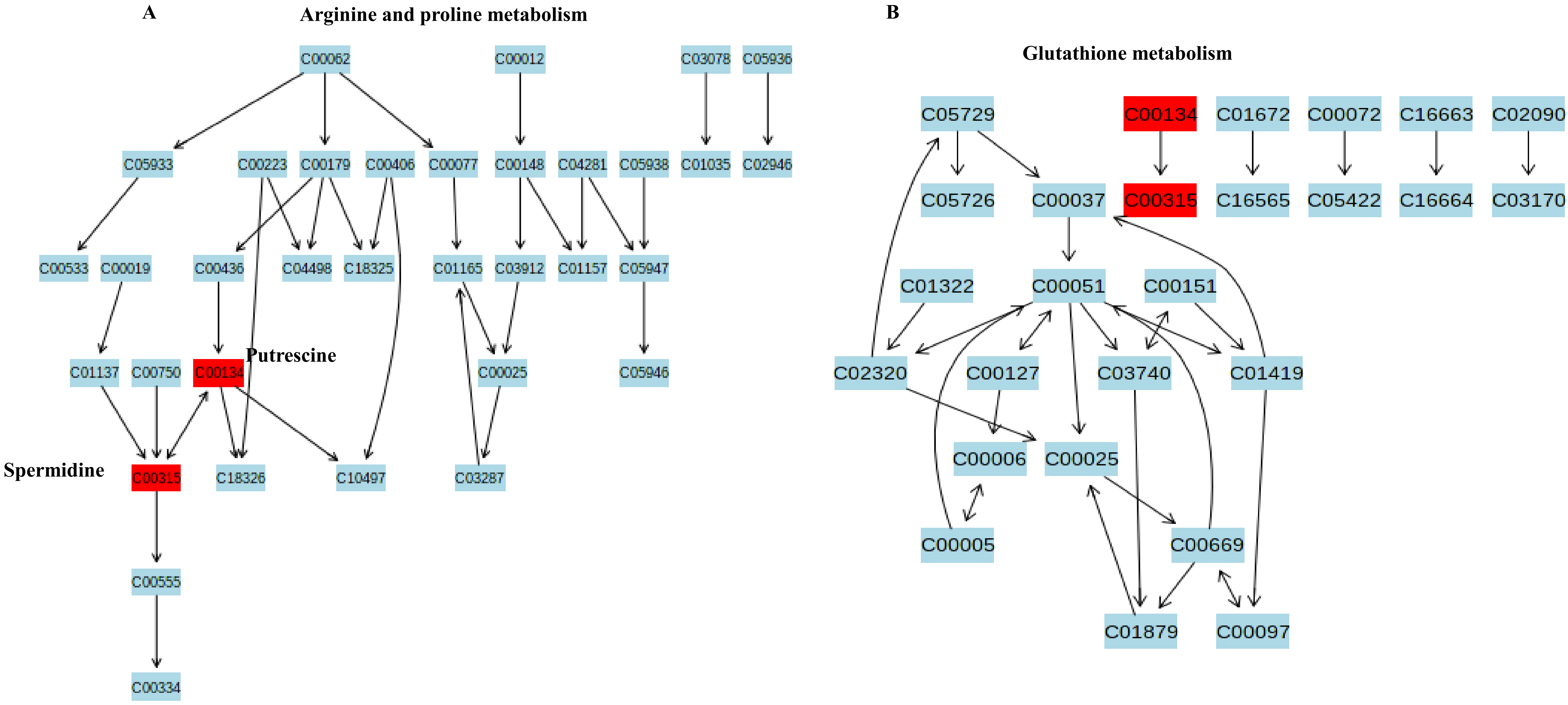


**Supplementary Figure S9 KEGG analyses of common down-regulated metabolites between M28 and K326 after drought treatment**

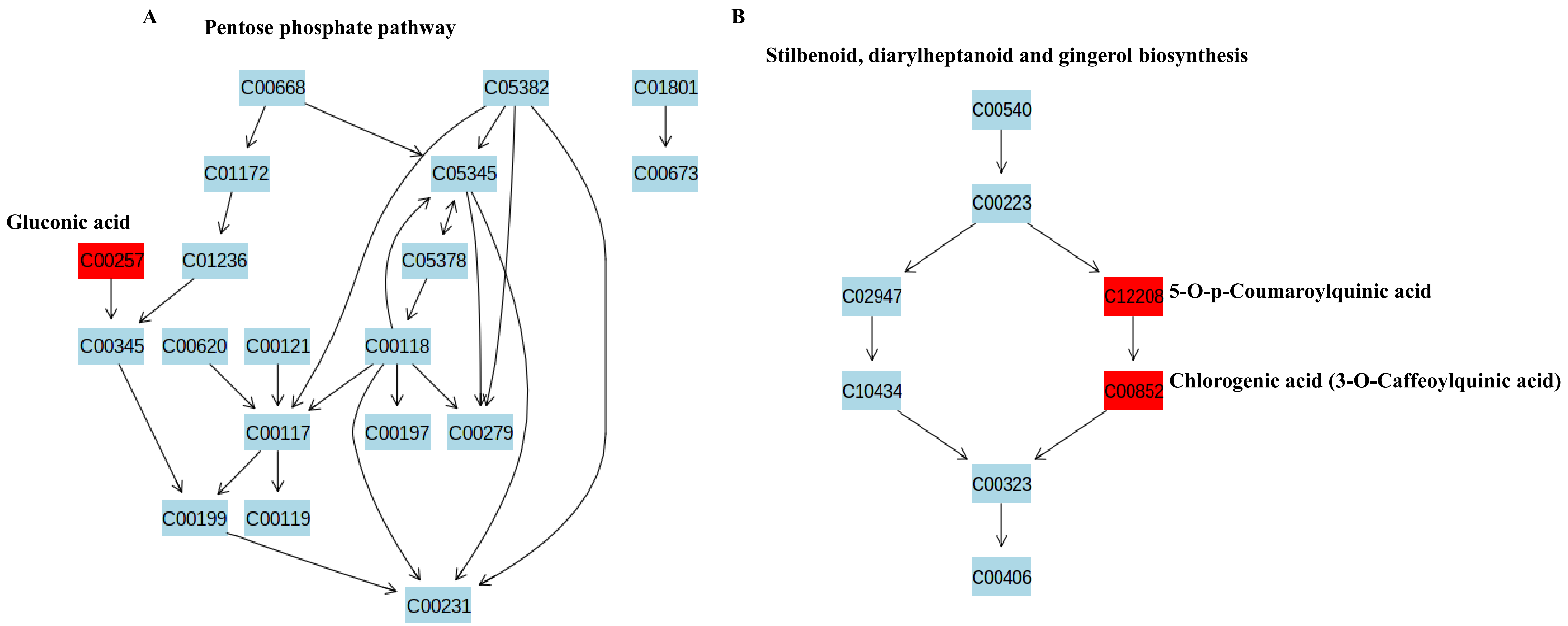


**Supplementary Figure S10 KEGG analyses of common up-regulated metabolites between M28 and K326 after drought treatment**

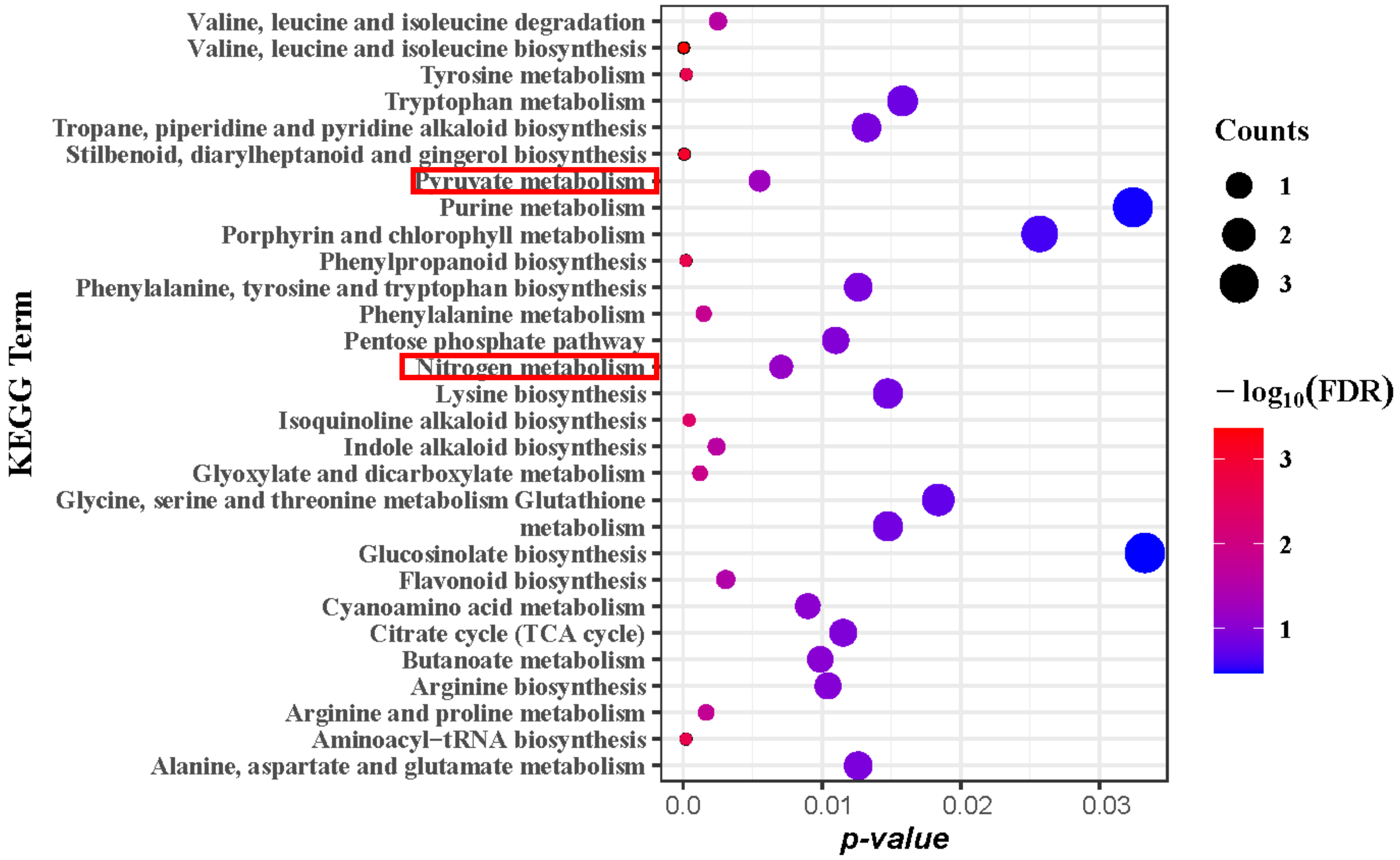


**Supplementary Figure S11 KEGG analyses for all drought induced DEGs.**

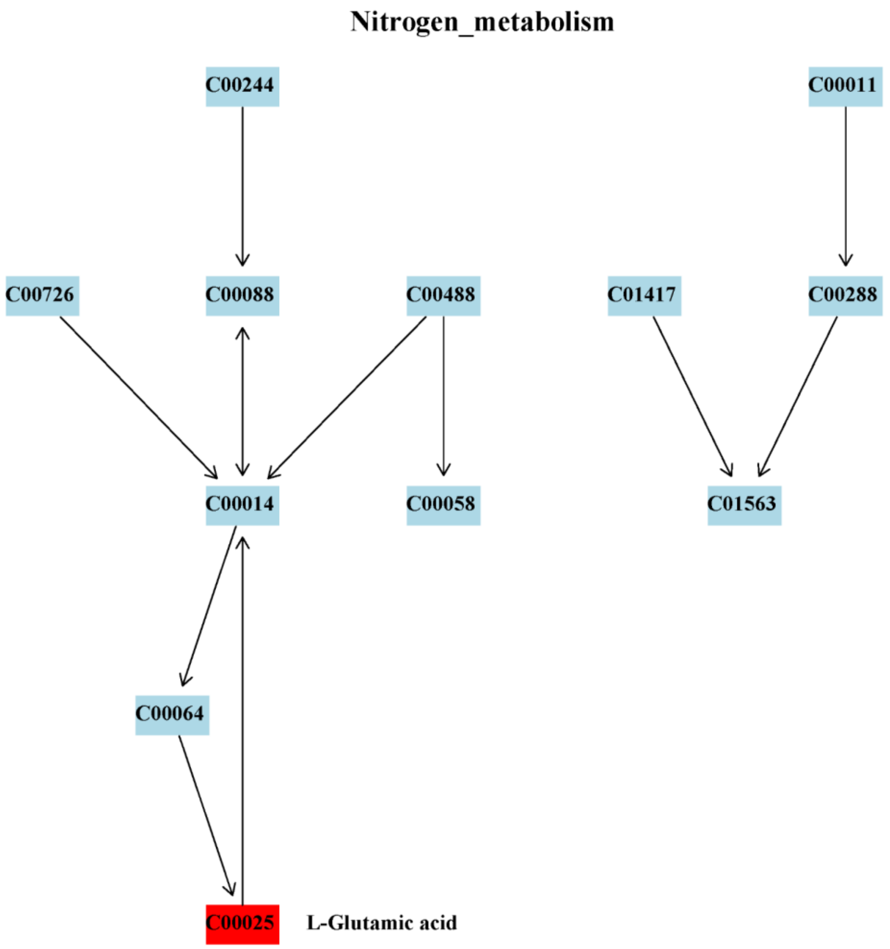


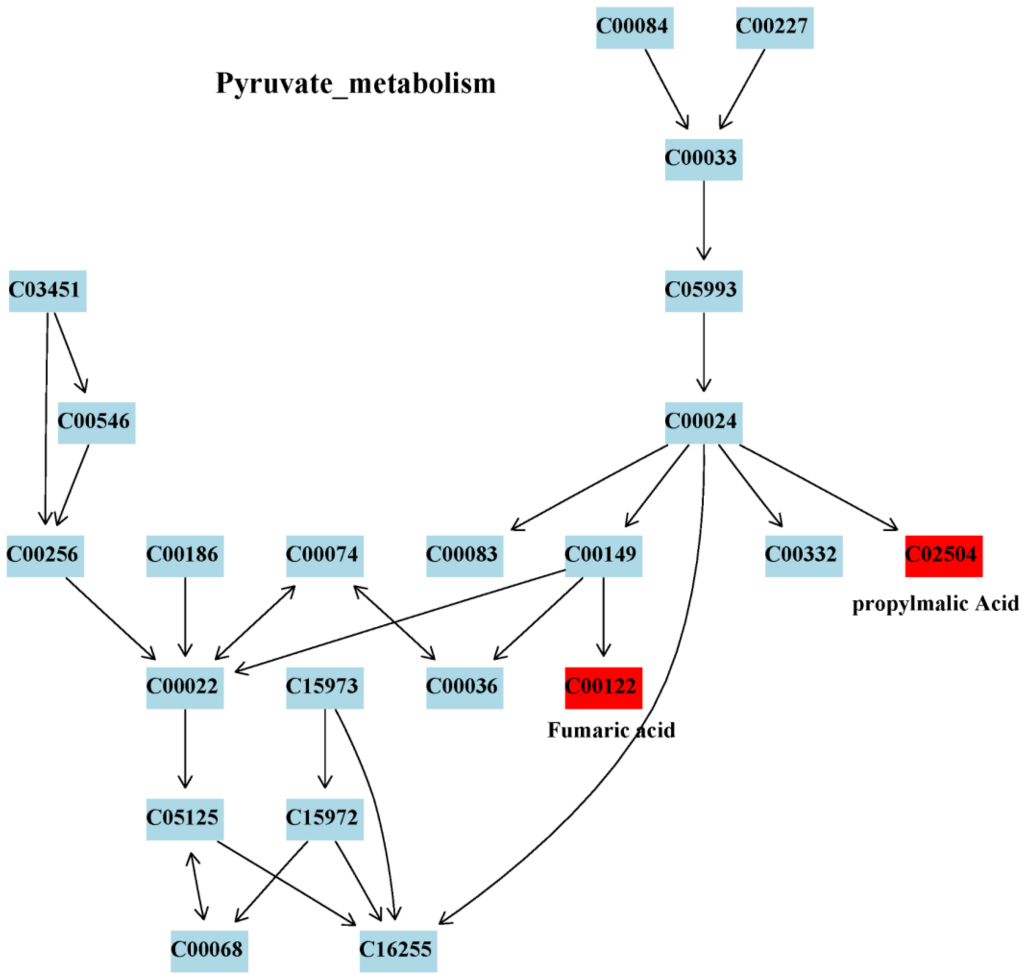


**Supplementary Figure S12 Overview of the KEGG pathway of Nitrogen_metabolism (left) and Pyruvate_metabolism (right).** The red indicates significantly up-regulated metabolites post drought treatment.

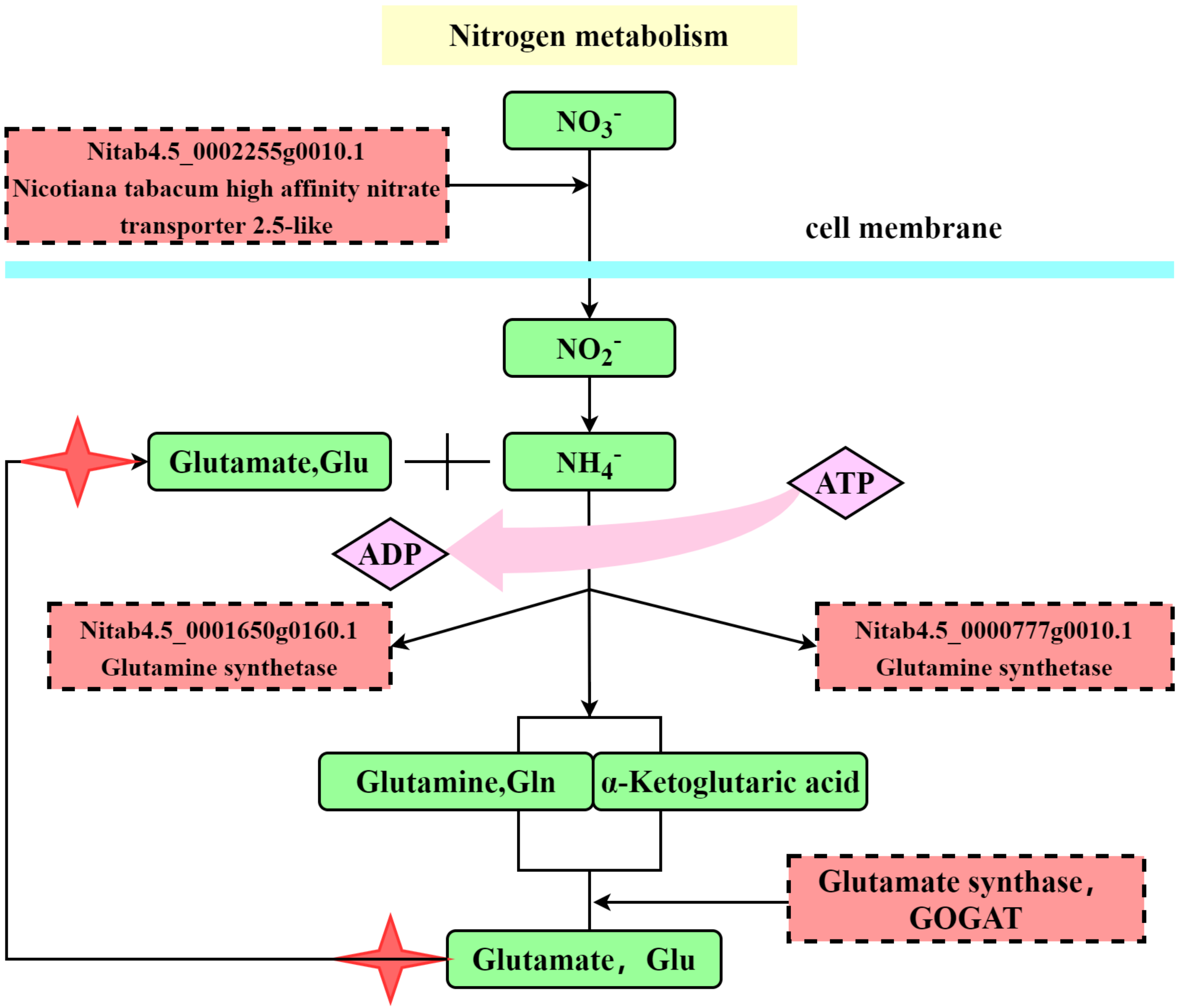


**Supplementary Figure S13 Involvement of DEGs in combination with metabolites in Nitrogen_metabolism pathway for synthesis of glutamate in tobacco.** The red representing DEGs and differential metabolites that were significantly enriched in the pathways of Nitrogen_metabolism .

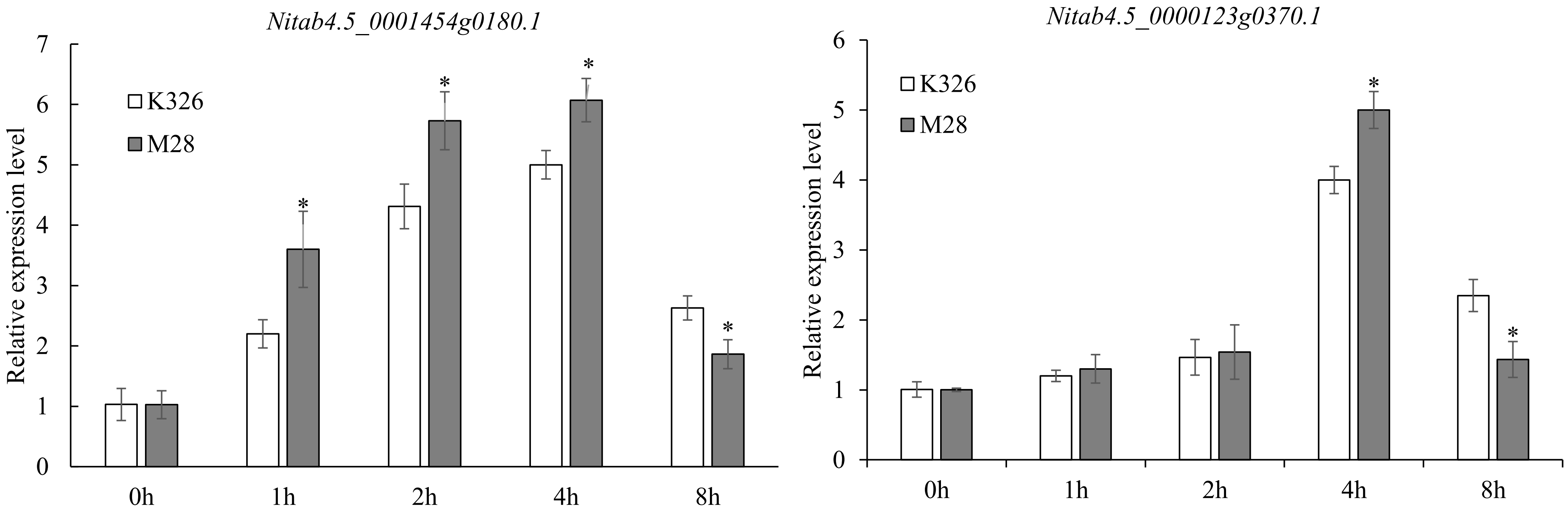


**Supplementary Figure S14 Relative expression levels of *Nitab4.5_0001454g0180* and *Nitab4.5_0000123g0370* in overview of DEGs and metabolites involved in "Phenylpropanoid biosynthesis" and "Flavonoid biosynthesis"**. Significance test was determined using t-test, *: 0.05 > p> 0.01; **: 0.01 > p> 0.001; ***: p<0.001.

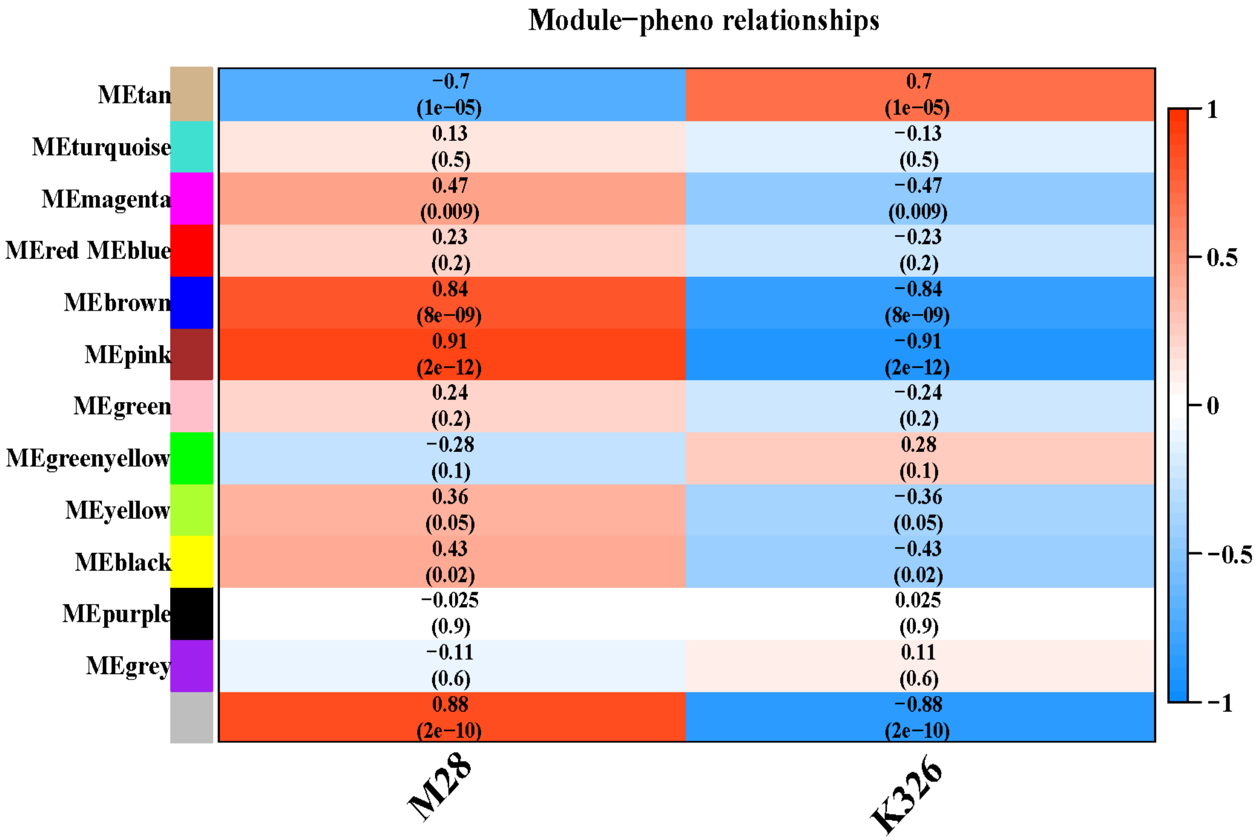


**Supplementary Figure S15 Module–sample association. Each row represents a module.** Each column corresponds to a specific sample. The color of each cell at the row–column intersection indicates the correlation coefficient between the module and sample. The red indicating a high degree of correlation between a specific module and sample.

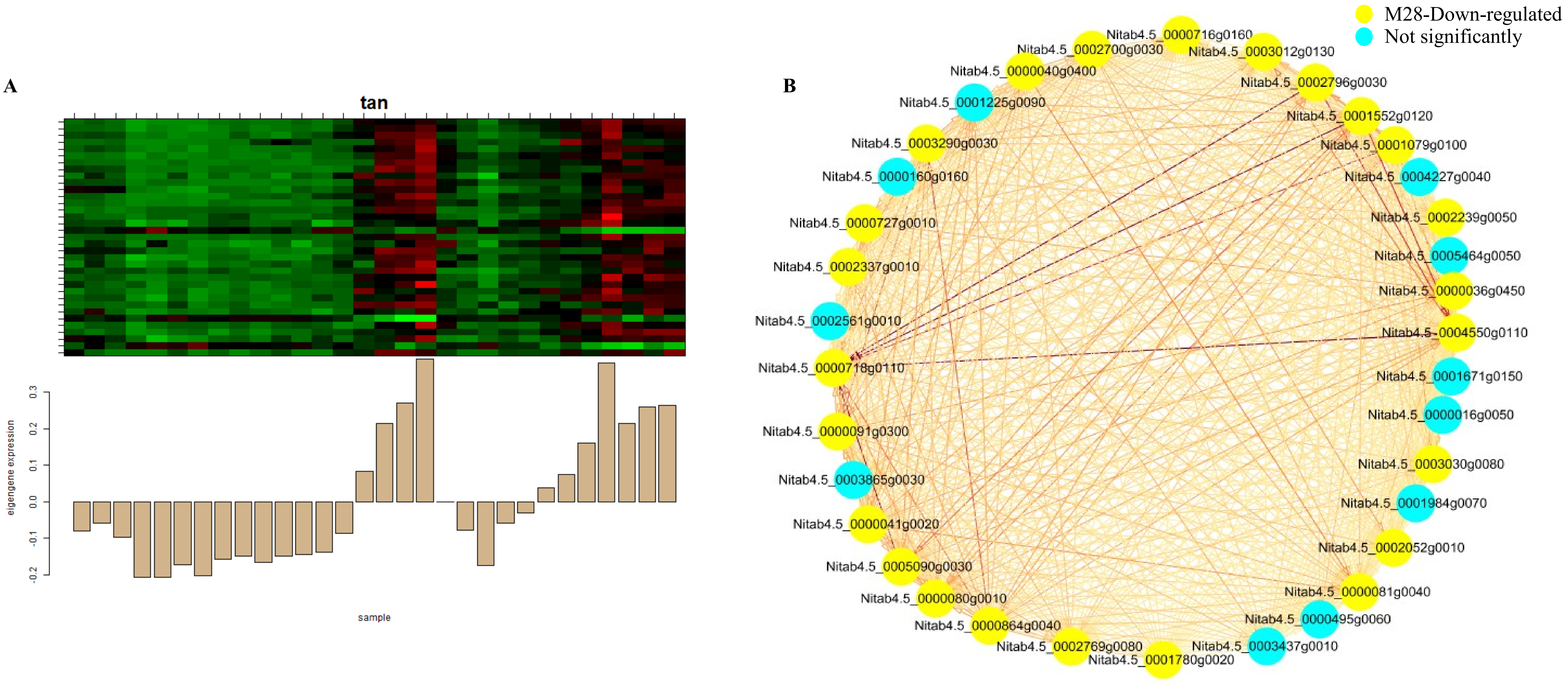


**Supplementary Figure S16 WGCNA analyses**. **(A)** Module Gene expression pattern in the tan module, the red representing up-regulated genes, the green representing down-regulated genes. **(B)** The correlation network of genes in the tan module. A gene network is constructed by WGCNA, in which each node represents a gene, and the connecting line between genes represents the co-expression correlation, the blue representing not significant genes and the yellow representing down-regulated genes between M28 and K326 before and after drought treatment . The genes with weights >0.1 are visualized by Cytoscape.

**Supplementary Table S1. Summary of time-point-related drought-induced DEGs in M28 or K326 or between M28 and K326**

| | DEGs | |
|---|---|---|
| | Down-regulated | Up-regulated |
| **28_0 vs K326_0** | 700 | 782 |
| **28_1 vs K326_1** | 503 | 502 |
| **28_2 vs K326_2** | 455 | 464 |
| **28_4 vs K326_4** | 521 | 557 |
| **28_8 vs K326_8** | 378 | 490 |
| **28_1 vs 28_0** | 358 | 529 |
| **28_2 vs 28_0** | 382 | 515 |
| **28_4 vs 28_0** | 776 | 966 |
| **28_8 vs 28_0** | 1,553 | 1,543 |
| **28_2 vs 28_1** | 41 | 58 |
| **28_4 vs 28_1** | 389 | 356 |
| **28_8 vs 28_1** | 1,224 | 1,135 |
| **28_4 vs 28_2** | 157 | 106 |
| **28_8 vs 28_2** | 1,072 | 918 |
| **28_8 vs 28_4** | 677 | 594 |
| **K326_1 vs K326_0** | 620 | 770 |
| **K326_2 vs K326_0** | 646 | 722 |
| **K326_4 vs K326_0** | 1,073 | 1,199 |
| **K326_8 vs K326_0** | 1,920 | 1,707 |
| **K326_2 vs K326_1** | 298 | 175 |
| **K326_4 vs K326_1** | 671 | 644 |
| **K326_8 vs K326_1** | 1,519 | 1,384 |
| **K326_4 vs K326_2** | 183 | 194 |
| **K326_8 vs K326_2** | 1,223 | 996 |
| **K326_8 vs K326_4** | 613 | 387 |

**Supplementary Table S2. Summary of time-point-related drought-induced DiffExp metabolites in M28 or K326 or between M28 and K326**

| | DiffExp metabolites statistics | |
|---|---|---|
| | **Down-regulated** | **Up-regulated** |
| **28_0 vs K326_0** | 34 | 36 |
| **28_1 vs K326_1** | 16 | 26 |
| **28_2 vs K326_2** | 51 | 21 |
| **28_4 vs K326_4** | 9 | 36 |
| **28_8 vs K326_8** | 20 | 19 |
| **28_1 vs 28_0** | 0 | 21 |
| **28_2 vs 28_0** | 15 | 11 |
| **28_4 vs 28_0** | 7 | 22 |
| **28_8 vs 28_0** | 2 | 21 |
| **28_2 vs 28_1** | 18 | 2 |
| **28_4 vs 28_1** | 16 | 6 |
| **28_8 vs 28_1** | 11 | 10 |
| **28_4 vs 28_2** | 1 | 8 |
| **28_8 vs 28_2** | 1 | 23 |
| **28_8 vs 28_4** | 10 | 7 |
| **K326_1 vs K326_0** | 4 | 9 |
| **K326_2 vs K326_0** | 2 | 69 |
| **K326_4 vs K326_0** | 10 | 11 |
| **K326_8 vs K326_0** | 5 | 38 |
| **K326_2 vs K326_1** | 1 | 17 |
| **K326_4 vs K326_1** | 3 | 0 |
| **K326_8 vs K326_1** | 4 | 23 |
| **K326_4 vs K326_2** | 43 | 0 |
| **K326_8 vs K326_2** | 20 | 13 |
| **K326_8 vs K326_4** | 1 | 10 |

**Supplementary Table S8. Up-regulated and Down-regulated genes in brown and Tan modules**

| up-regulated genes in brown module | down-regulated genes in Tan module |
|---|---|
| Nitab4.5_0000040g0380 | Nitab4.5_0000036g0450 |
| Nitab4.5_0000050g0020 | Nitab4.5_0000040g0400 |
| Nitab4.5_0000062g0300 | Nitab4.5_0000041g0020 |
| Nitab4.5_0000092g0390 | Nitab4.5_0000080g0010 |
| Nitab4.5_0000110g0090 | Nitab4.5_0000081g0040 |
| Nitab4.5_0000117g0150 | Nitab4.5_0000091g0300 |
| Nitab4.5_0000173g0140 | Nitab4.5_0000716g0160 |
| Nitab4.5_0000178g0210 | Nitab4.5_0000718g0110 |
| Nitab4.5_0000195g0170 | Nitab4.5_0000727g0010 |
| Nitab4.5_0000303g0130 | Nitab4.5_0000864g0040 |
| Nitab4.5_0000458g0100 | Nitab4.5_0001079g0100 |
| Nitab4.5_0000500g0090 | Nitab4.5_0001552g0120 |
| Nitab4.5_0000525g0020 | Nitab4.5_0001780g0020 |
| Nitab4.5_0000856g0240 | Nitab4.5_0002052g0010 |
| Nitab4.5_0000978g0010 | Nitab4.5_0002239g0050 |
| Nitab4.5_0000993g0090 | Nitab4.5_0002337g0010 |
| Nitab4.5_0001315g0150 | Nitab4.5_0002700g0030 |
| Nitab4.5_0001315g0190 | Nitab4.5_0002769g0080 |
| Nitab4.5_0001925g0020 | Nitab4.5_0002796g0030 |
| Nitab4.5_0001996g0010 | Nitab4.5_0003012g0130 |
| Nitab4.5_0002367g0080 | Nitab4.5_0003030g0080 |
| Nitab4.5_0002682g0080 | Nitab4.5_0003290g0030 |
| Nitab4.5_0003286g0020 | Nitab4.5_0004550g0110 |
| Nitab4.5_0004274g0040 | Nitab4.5_0005090g0030 |
| Nitab4.5_0005820g0010 | |
| Nitab4.5_0005901g0010 | |

**Supplementary Table S9. Blue module contained 15 up-regulated genes and 3 TFs**

| up-regulated genes | TFs |
|---|---|
| Nitab4.5_0000175g0070 | *bHLH* |
| Nitab4.5_0000540g0010 | *C3H* |
| Nitab4.5_0000082g0020 | *CCAAT* |
| Nitab4.5_0000986g0060 | up-regulated genes |
| Nitab4.5_0001788g0010 | up-regulated genes |
| Nitab4.5_0004624g0020 | up-regulated genes |
| Nitab4.5_0000312g0130 | up-regulated genes |
| Nitab4.5_0005745g0030 | up-regulated genes |
| Nitab4.5_0002887g0100 | up-regulated genes |
| Nitab4.5_0002079g0020 | up-regulated genes |
| Nitab4.5_0000142g0080 | up-regulated genes |
| Nitab4.5_0000832g0190 | up-regulated genes |
| Nitab4.5_0002093g0090 | up-regulated genes |
| Nitab4.5_0000471g0030 | up-regulated genes |
| Nitab4.5_0000130g0300 | up-regulated genes |
| Nitab4.5_0001239g0040 | up-regulated genes |
| Nitab4.5_0002716g0020 | up-regulated genes |
| Nitab4.5_0000463g0070 | up-regulated genes |

**Supplementary Table S10. Primers used for gene expression analyses**

| Gene name | Primer | Sequence |
|---|---|---|
| *NCED1* | F | GGCCATTGCAGAACCATGGCCAAA |
| | R | GCTTTGCTGTTAGGGTCTCTTGG |
| *PP2C-37L* | F | ACCCGTCGTTTTGCAAAGGAG |
| | R | ACCTCTTTCCACTGAGTTTCCCC |
| *P450 84A1L* | F | GGGACAGCTGTTAACTTAGG |
| | R | TAGACTCTGCTTACCAACCCAC |
| *18srDNA* | F | AGGATTGACAGACTGAGAGC |
| | R | CACAGACCTGTTATTGCCTC |
| *Nitab4.5_0001454g0180.1* | F | GCGAGTGTTCTTCACATCCTAAA |
| | R | GCAGAAGGAGTCTCGTAGAGAAGT |
| *Nitab4.5_0000123g0370.1* | F | TCCAATGTGTAGCCAAGGAAGG |
| | R | ACGGCTGGATCACGAGCAACA |
